# Cerebrospinal Fluid and Blood Cytokines as Biomarkers for Multiple Sclerosis: A Systematic Review and Meta-Analysis of 226 Studies With 13,526 Multiple Sclerosis Patients

**DOI:** 10.3389/fnins.2019.01026

**Published:** 2019-10-04

**Authors:** Zhile Bai, Duanduan Chen, Luyao Wang, Yu Zhao, Tiantian Liu, Yun Yu, Tianyi Yan, Yong Cheng

**Affiliations:** ^1^Center on Translational Neuroscience, College of Life and Environmental Sciences, Minzu University of China, Beijing, China; ^2^School of Life Science, Beijing Institute of Technology, Beijing, China; ^3^School of Mechatronical Engineering, Beijing Institute of Technology, Beijing, China

**Keywords:** cytokines, multiple sclerosis, peripheral blood, cerebrospinal fluid, meta-analysis, systematic review

## Abstract

**Background:** Multiple sclerosis (MS) biomarker identification is important for pathogenesis research and diagnosis in routine clinical practice. Cerebrospinal fluid (CSF) and blood cytokines as potential biomarkers that can inform MS pathogenesis, diagnosis and response to treatment have been assessed in numerous studies. However, there have been no comprehensive meta-analyses to pool cytokine data and to address their diagnostic performance. We systematically reviewed literature with meta-analyses to assess the alteration levels of cytokines and chemokines in MS.

**Methods:** We searched PubMed and Web of Science for articles published between January 1, 1990 and April 30, 2018 for this systematic review and meta-analysis. Data were extracted from 226 included studies encompassing 13,526 MS patients and 8,428 controls. Biomarker performance was rated by a random-effects meta-analysis based on the standard mean difference between cytokine concentration in patients with MS and controls, or patients before and after treatments.

**Results:** Of the 26 CSF cytokines and 37 blood cytokines for potential differentiation between MS patients and controls, the random-effects meta-analysis showed that 13 CSF cytokines and 21 blood cytokines were significantly increased in MS patients in comparison to the controls. Interestingly, TNF-α, CXCL8, IL-15, IL-12p40, and CXCL13 were increased in both blood and CSF of MS patients. For those cytokines analyzed in at least 10 studies, differentiation between case and control was strong for CSF CXCL13, blood IL-2R, and blood IL-23; CSF CXCL8, blood IL-2, and blood IL-17 also performed well in differentiating between MS patients and controls, whereas those of CSF TNF-α and blood TNF-α, CXCL8, IL-12, IFN-γ were moderate. Furthermore, CSF IL-15, CCL19, CCL11, CCL-3, and blood CCL20, IL-12p40, IL-21, IL-17F, IL-22 had large effective sizes when differentiating between MS patients and controls but had a relatively small number of studies (three to seven studies).

**Conclusion:** Our findings clarified the circulating cytokine profile in MS, which provide targets for disease modifying treatments, and suggest that cytokines have the potential to be used as biomarkers for MS.

## Introduction

Multiple sclerosis (MS) is the most common autoimmune disease that affects the central nervous system (Berer and Krishnamoorthy, 2014). The prevalence of MS varies greatly in different regions and it was found to be significantly associated with latitude (Simpson et al., 2011). There are several forms of the disease and ~85% of cases are presented as relapsing-remitting MS (RRMS) initially, and most RRMS patients convert to secondary progressive MS (SPMS) after 10–20 years of disease progression, while the remaining 15% of cases experience a primary progressive disease course (PPMS) (Sartori et al., 2017; Bonin et al., 2018). Although the underlying mechanism for MS is believed to be either an immune system dysfunction or the neurodegeneration of myelin-producing cells (Schreiner and Becher, 2015), the cause for the destruction of the immune system or neurodegeneration is currently poorly understood. Currently, there is no treatment to cure the disease, and the traditional first-line medications for treatments of MS are interferon-beta and glatiramer acetate with moderate effects; and a substantial number of patients fail to respond to these treatments (Mahurkar et al., 2014). However, more recently developed drugs such as natalizumab, alemtuzumab, and ocrelizumab have shown to effectively slow the disease progression down for some MS patients. Nevertheless, there is a need to better understand the etiology of MS and to develop disease modifying treatments.

Although the primary nature of MS pathogenesis is still under debate, overall evidence from pre-clinical and clinical studies overwhelmingly support the concept that MS commences in the immune system and that the demyelination of the central nervous system is the wrong target of the immune attack (Schreiner and Becher, 2015). In fact, the earlier predominant view was that MS is a disease driven by Th1 cells which produce high levels of pro-inflammatory cytokines including IFN-γ and IL-12, and the preponderance of Th1 over Th2 cells contributed to the shift toward a pro-inflammatory profile in patients during MS relapse (Steinman, 2007). However, more recent studies have emphasized a critical role of Th17 cells in neuroinflammation and MS pathogenesis, which involves the aberrations of cytokine IL-17 and IL-23 in the disease (Luchtman et al., 2014). Nevertheless, a cytokine-mediated inflammatory response is believed to be a key process of the autoimmune attack, and the derailed immune communication orchestrated by the cytokines provide attractive targets for the immunotherapy of MS (Schreiner and Becher, 2015). In fact, research on the role of cytokines in MS has exploded over the last several decades, in the hope of gaining insight into the pathogenesis of MS and providing biomarkers for the diagnosis, prognosis, and response to drug treatment, and eventually in developing disease modifying treatments (Amedei et al., 2012; Fitzner et al., 2015; Yadav et al., 2015). Indeed, a large number of clinical studies showed increased pro-inflammatory cytokines in the blood and cerebrospinal fluid (CSF) of patients with MS, these cytokines include TNF-α, IL-17, CXCL8, IL-17, IL-23, and CXCL-13 (Drulovic et al., 1997, 1998; Lund et al., 2004; Ragheb et al., 2011; Alvarez et al., 2013; Huber et al., 2014; Babaloo et al., 2015; Farrokhi et al., 2015; Salehi et al., 2016; Bonin et al., 2018). However, the clinical data for the significant associations between circulating cytokines and MS were inconsistent for individual cytokines and between studies.

To analyze CSF and blood cytokine aberrations in MS patients, we systematically searched the literature and performed meta-analyses to allow data from individual studies to be pooled quantitatively, to strengthen the clinical data of inflammatory cytokine profile in MS.

## Materials and Methods

The systematic review and meta-analysis performed in this study followed guidelines that are recommended by the PRISMA statement (Preferred Reporting Items for Systematic Reviews and Meta-analysis; Moher et al., 2009).

### Search Strategy and Study Selection

The systematic review of English-language articles was performed by five independent investigators from the databases of PubMed and Web of Science between January 1, 1990 and April 30, 2018. The search term was: (inflammation or cytokine or chemokine or tumor necrosis factor or interleukin or interferon or C-reactive protein) AND multiple sclerosis. Cross sectional studies reporting data on blood or CSF cytokine concentrations in patients with MS and controls were included, and longitudinal studies analyzing circulating cytokine changes for drug treatments in MS patients were also included. Controls included healthy control subjects and patients with other diseases. It should be noted that many CSF studies used patients with other diseases as controls because it was difficult to obtain CSF samples from healthy subjects, whereas only a few blood studies used patients with other diseases as controls. The exclusion criteria were: (1) *in vitro* studies which reported stimulated or unstimulated levels of cytokines; (2) same patient samples with other studies; (3) samples were taken after patients died; (4) Cytokines were assessed in <3 studies; (5) without a control group.

### Data Extraction

The data were extracted by two investigators and checked by another two investigators. We extracted sample size, mean cytokine concentrations with standard deviation (s.d.) and *P*-values from the included studies as the primary outcomes. Data on age, gender, publication year, disease severity (Expanded Disability Status Scale, EDSS), MS subtype, country, sampling source, assay type, and medication status were also extracted for potential moderator analyses (Supplementary Material eTable 1). The medication included treatments with IFN-beta, methylprednisolone, natalizumab, fingolimod and rituximab. The quality of the studies was assessed by the Newcastle-Ottawa quality assessment scale (Supplementary Material eTable 2).

### Statistical Analysis

The Comprehensive Meta-analysis software (version 2; Biostat Inc.) was used to perform all the statistical analyses in this study. Sample size and mean cytokine concentration with s.d. were primarily used to generate effective sizes (ESs), and ESs were generated by sample size and *P*-value if cytokine concentration data were not available. ESs were calculated by standardized mean differences (SMD) in cytokine concentrations between patients with MS and the controls, or before and after drug treatment for MS patients. We calculated an ES estimate for each blood or CSF cytokine analyzed in this meta-analysis. The random-effects meta-analysis was chosen for this study because we hypothesized that both between-study and within-study variances affected the true ES.

We used the Cochrane Q test and I^2^ statistic to assess between-study heterogeneity, and I^2^ statistics of 0.25, 0.5, and 0.75 are considered to be small, moderate, and high levels of heterogeneity, respectively (Qin et al., 2016). We performed unrestricted maximum-likelihood random-effects meta-regressions to analyze whether the outcomes of the meta-analysis were affected by the continuous variables, including age, gender (proportion of male subjects), and disease severity. In addition, the Egger test was used to assess the publication bias.

*P*-values less than 0.05 were considered statistically significant in all the analyses except for the Cochrane *Q*-test, the statistical significance of which was set for *P* < 0.1.

## Results

**Figure 1:**
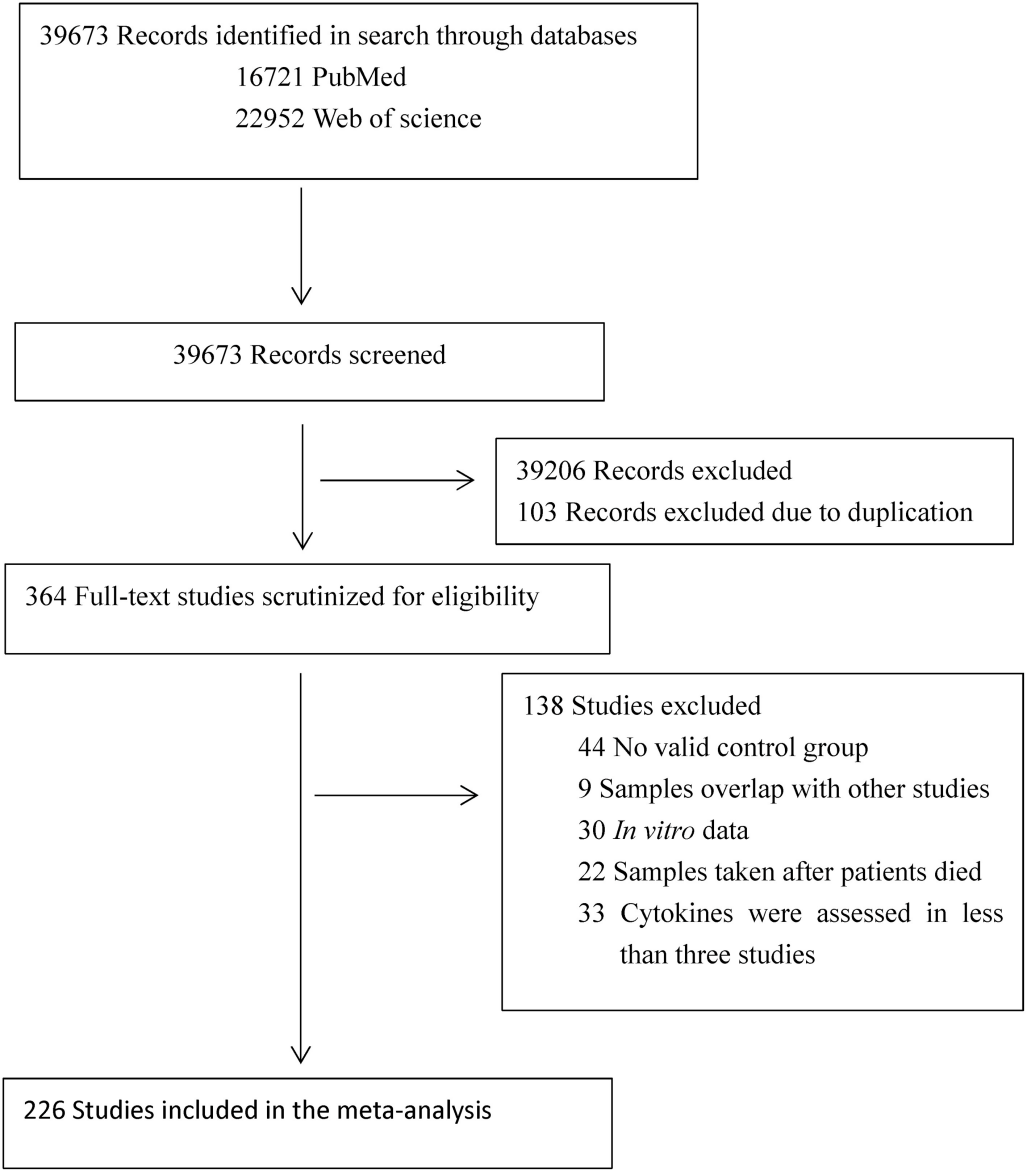
PRISMA flowchart of the literature search.

The initial keyword search identified 16,721 records from PubMed and 22,952 records from Web of Science. After screening the titles and abstracts, 364 articles were selected for full text scrutiny. Of the 364 articles, 138 Studies were excluded due to: not having a control group (44 studies); samples overlapped with other studies (9 studies); studies with *in vitro* cytokine data (30 studies); samples were taken after patients died (22 studies); cytokines were assessed in <3 studies (33 studies). Therefore, a total of 226 articles (eReference in the Supplementary Materials) comprising 13,526 MS patients and 8,428 controls were included in this meta-analysis (Flowchart see Figure 1).

### Main Associations of MS With Blood Cytokines

**Figure 2:**
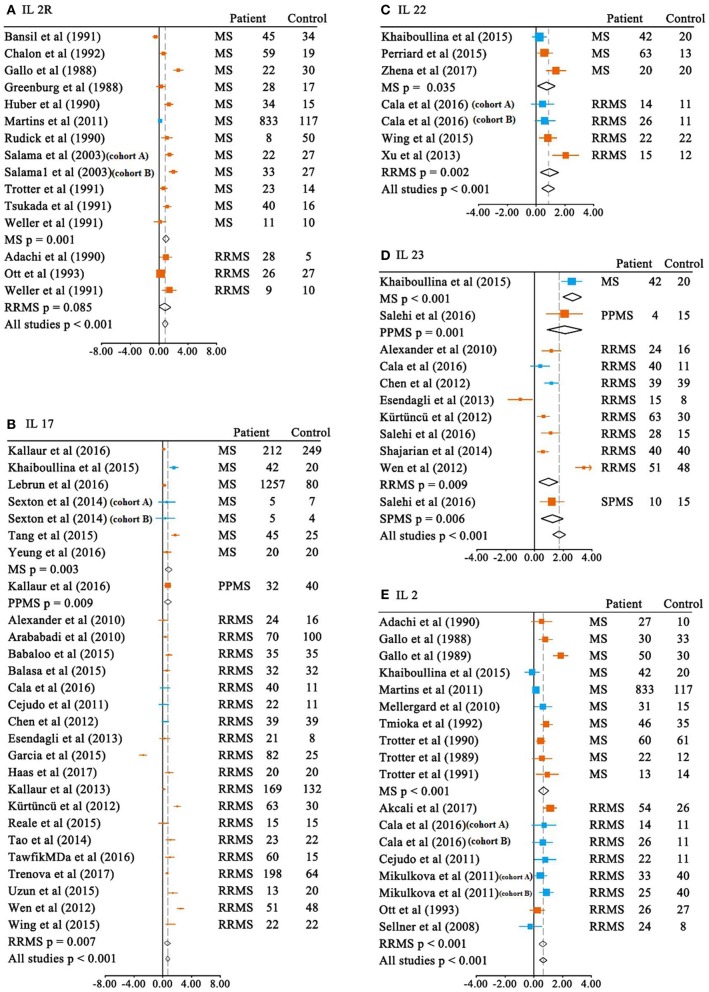
Multiple sclerosis to control SMD for blood cytokines. Blood SMDs of IL-2R **(A)**, IL-17 **(B)**, IL-22 **(C)**, IL-23 **(D)**, IL-2 **(E)** between multiple sclerosis and controls. Note that the subtypes of the MS are presented in the figures unless the included studies in the meta-analysis did not provide the information. MS, multiple sclerosis; RRMS, relapsing-remitting MS; PPMS, primary progressive MS; SPMS, secondary progressive MS; SMD, standardized mean difference; 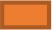 ELISA; 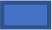 non-ELISA.

We first meta-analyzed data for the blood cytokine differences between MS patients and the controls. Of the 37 blood cytokines analyzed in the meta-analysis, 21 cytokine levels were significantly increased in patients with MS when compared with the control subjects (Table 1A and Figure 2). For those blood cytokines significantly associated with MS, CCL20, IL-23, IL-21, IL-12p40, IL17F, IL22, and IL2R had large ESs to differentiate between MS patients and the controls (SMD 0.820–2.229). Blood IL-15, IL-2, IL-17, IL-33, and IL-16 also had good performance to differentiate between cases and controls (SMD 0.614–0.784), whereas those of TNF-α, IL-12, IL-13, CCL-5, CXCL13, IL-12p70, CXCL12, IFN-γ, and CXCL8 were moderate (SMD 0.244–0.498).

**Table 1:** Summary of cytokine data in multiple sclerosis.

**Cytokine**	**No. of studies**	**No. with MS/controls**	**Main effect**			**Heterogeneity**				**Publication bias**	
			**Std diff in means (95% CI)**	***Z* score**	***P*-value**	**Q statistic**	**df**	***P*-value**	**I^∧^2 statistic**	**Egger intercept**	***P*-value**
**A. SUMMARY OF BLOOD CYTOKINE LEVELS BETWEEN CASES AND CONTROLS**											
TNF-α	33	2,424/1,468	0.498	3.853	0.000	435.668	32	0.000	92.655	1.47543	0.28956
			(0.017 to 0.751)								
IL-6	28	1,667/795	0.209	1.854	0.064	169.474	27	0.000	84.068	−0.40943	0.67606
			(−0.012 to 0.430)								
IL-17	27	2,617/1,110	0.622	4.724	0.000	290.245	26	0.000	91.042	2.0724	0.13284
			(0.364 to 0.881)								
IFN-γ	26	1,837/956	0.267	3.994	0.000	114.190	25	0.000	78.107	2.25901	0.01011
			(0.136 to 0.397)								
IL-10	24	1,799/885	0.086	1.063	0.288	140.521	23	0.000	83.632	0.93155	0.40090
			(−0.073 to 0.245)								
CXCL8	18	1,441/647	0.244	2.343	0.019	83.265	17	0.000	79.583	1.40273	0.23444
			(0.040 to 0.448)								
IL-2	18	1,378/470	0.630	5.614	0.000	61.940	17	0.000	72.554	1.99978	0.05989
			(0.410 to 0.850)								
IL-2R	15	1,299/381	0.820	3.824	0.000	114.146	14	0.000	87.735	3.47917	0.01713
			(0.391 to 1.213)								
IL-4	13	1,486/636	0.209	1.797	0.072	69.128	13	0.000	81.194	−1.12494	0.39067
			(−0.019 to 0.436)								
IL-1β	12	1,462/613	0.194	1.523	0.128	58.405	11	0.000	81.166	−0.35463	0.80153
			(−0.055 to 0.443)								
IL-23	11	356/227	1.711	8.100	0.000	108.636	10	0.000	90.795	1.74042	0.65065
			(1.297 to 2.125)								
CCL2	11	471/227	−0.060	−0.363	0.717	91.304	10	0.000	89.048	5.44798	0.07713
			(−0.384 to 0.264)								
CRP	10	570/457	0.602	1.597	0.110	160.128	9	0.000	94.379	2.51736	0.37491
			(−0.137 to 1.342)								
IL-12	10	481/382	0.490	2.330	0.020	186.900	9	0.000	95.185	5.03348	0.08092
			(0.078 to 0.902)								
CCL5	7	318/120	0.443	3.250	0.001	29.957	6	0.000	79.972	2.09314	0.53193
			(0.176 to 0.709)								
IL-5	7	999/192	−0.023	−0.168	0.867	12.657	6	0.049	52.595	−0.68231	0.57266
			(−0.291 to 0.245)								
IL-22	7	202/98	0.821	3.647	0.000	15.458	6	0.017	61.186	6.2265	0.07951
			(0.380 to 1.262)								
CCL11	6	219/130	0.095	0.335	0.737	31.111	5	0.000	83.929	1.85402	0.61518
			(−0.462 to 0.653)								
IL-13	6	1,026/225	0.485	3.689	0.000	13.058	5	0.023	61.709	2.29043	0.08742
			(0.227 to 0.742)								
IL-12P70	6	218/124	0.328	2.066	0.039	7.128	5	0.211	29.852	−2.89296	0.21611
			(0.017 to 0.639)								
TGF-β1	6	194/108	0.377	1.843	0.065	42.308	5	0.000	88.182	−2.37852	0.64912
			(−0.024 to 0.779)								
CCL4	5	213/108	−0.005	−0.015	0.988	33.006	4	0.000	87.881	4.17416	0.42539
			(−0.732 to 0.722)								
IL-33	5	298/247	0.614	4.100	0.000	288.211	4	0.000	98.612	−1.33406	0.9681
			(0.320 to 0.907)								
IL-21	5	131/79	1.188	3.777	0.000	13.266	4	0.010	69.849	7.37721	0.0149
			(0.571 to 1.804)								
BAFF	5	313/115	−0.016	−0.066	0.947	33.577	4	0.000	88.087	−4.59612	0.38674
			(−0.481 to 0.450)								
CCL20	4	249/173	2.229	14.286	0.000	237.888	3	0.000	98.739	−11.96333	0.66858
			(1.923 to 2.536)								
CXCL13	4	100/73	0.420	2.664	0.008	1.066	3	0.785	0.000	−0.09159	0.96557
			(0.111 to 0.728)								
IL-7	4	292/92	−0.130	−0.454	0.650	11.774	3	0.008	74.521	8.04502	0.05458
			(−0.693 to 0.432)								
IL-12P40	4	121/89	1.269	4.530	0.000	46.205	3	0.000	93.507	8.70515	0.25968
			(0.720 to 1.818)								
IL-15	4	158/72	0.784	5.244	0.000	1.096	3	0.778	0.000	−4.87584	0.18103
			(0.491 to 1.077)								
IL-17F	4	140/88	0.981	6.602	0.000	4.987	3	0.173	39.840	−0.10914	0.98794
			(0.690 to 1.272)								
IL-18	4	244/210	0.154	1.089	0.276	15.146	3	0.002	80.192	5.33867	0.16873
			(−0.124 to 0.432)								
CXCL12	3	156/127	0.276	2.262	0.024	1.325	2	0.516	0.000	−0.61066	0.74177
			(0.037 to 0.514)								
IL-1α	3	72/43	0.158	0.648	0.517	3.035	2	0.219	34.095	2.21328	0.67187
			(−0.320 to 0.635)								
IL-9	3	145/61	0.174	0.688	0.492	15.305	2	0.000	86.932	12.49871	0.33727
			(−0.321 to 0.668)								
IL-16	3	308/437	0.605	3.047	0.002	56.255	2	0.000	96.445	−3.37926	0.71202
			(0.216 to 0.995)								
IL-27	3	125/76	−0.143	−0.811	0.417	15.787	2	0.000	87.332	4.31443	0.78931
			(−0.487 to 0.202)								
**B. SUMMARY OF CSF CYTOKINE LEVELS BETWEEN CASES AND CONTROLS**											
CXCL13	23	1,005/268	0.805	11.903	0.000	32.441	22	0.070	32.185	3.18745	0.00310
			(0.672 to 0.937)								
IL-6	20	551/501	0.116	1.397	0.162	60.350	19	0.000	68.517	0.80032	0.46866
			(−0.047 to 0.279)								
CXCL8	17	661/331	0.553	4.439	0.000	175.329	16	0.000	90.874	3.96989	0.14319
			(0.309 to 0.798)								
TNF-α	15	401/263	0.381	2.029	0.042	223.044	14	0.000	93.723	3.12681	0.37336
			(0.013 to 0.750)								
CCL2	12	354/193	−0.043	−0.302	0.763	57.487	11	0.000	80.865	−2.31561	0.43483
			(−0.323 to 0.237)								
IL-17	10	1,553/340	0.383	1.901	0.057	61.262	9	0.000	85.309	0.58837	0.77863
			(−0.012 to 0.777)								
CCL5	7	224/91	0.180	1.138	0.255	15.019	6	0.020	60.052	1.28612	0.70449
			(−0.130 to 0.490)								
IL-10	7	219/105	0.494	2.229	0.026	21.188	6	0.002	71.683	7.51938	0.08851
			(0.060 to 0.928)								
CCL19	6	103/55	0.899	4.055	0.000	7.104	5	0.213	29.622	0.28819	0.91316
			(0.464 to 1.333)								
IFN-γ	6	1,208/452	0.223	1.585	0.113	15.114	5	0.010	66.918	1.74427	0.67919
			(−0.053 to 0.498)								
CCL4	5	119/54	0.310	1.881	0.060	6.938	4	0.139	42.344	5.85423	0.02450
			(−0.013 to 0.633)								
CCL22	5	167/56	0.762	5.034	0.000	1.270	4	0.866	0.000	0.57686	0.70189
			(0.466 to 1.059)								
CXCL1	5	151/58	0.494	3.259	0.001	15.413	4	0.004	74.048	4.60918	0.32300
			(0.197 to 0.791)								
IL-12P40	5	213/113	0.545	4.165	0.000	6.216	4	0.184	35.653	4.42359	0.15018
			(0.288 to 0.801)								
BAFF	4	158/82	−0.269	−1.341	0.180	10.020	3	0.018	70.059	−5.81937	0.56641
			(−0.663 to 0.124)								
CCL11	4	91/61	0.834	2.671	0.008	10.147	3	0.017	70.436	−0.92717	0.90433
			(0.222 to 1.447)								
CXCL12	4	48/29	0.567	2.564	0.010	3.328	3	0.344	9.869	−7.90597	0.62992
			(0.134 to 1.000)								
IL-1β	4	157/81	0.192	0.908	0.364	44.351	3	0.000	93.236	−12.04068	0.34305
			(−0.222 to 0.606)								
IL-2	4	145/77	0.110	0.224	0.823	29.601	3	0.000	89.865	2.05241	0.88052
			(−0.851 to 1.070)								
IL-5	4	137/57	0.205	1.356	0.175	1.931	3	0.587	0.000	0.08847	0.97391
			(−0.091 to 0.502)								
IL-9	4	192/98	0.196	1.515	0.130	1.192	3	0.755	0.000	−0.68671	0.51596
			(−0.058 to 0.450)								
IL-15	4	137/48	0.921	3.686	0.000	14.783	3	0.002	79.707	5.49319	0.46068
			(0.431 to 1.410)								
sIL-6R	4	76/27	0.228	1.076	0.282	39.860	3	0.000	92.474	11.9736	0.13815
			(−0.187 to 0.644)								
CCL3	3	74/46	0.820	2.450	0.014	11.079	2	0.004	81.948	7.28863	0.24801
			(0.164 to 1.477)								
CCL21	3	35/24	0.959	1.969	0.049	13.367	2	0.001	85.038	10.91872	0.27285
			(0.004 to 1.914)								
IL-4	3	119/62	0.063	0.220	0.826	22.505	2	0.000	91.113	14.13443	0.34178
			(−0.499 to 0.625)								
**C. SUMMARY OF BLOOD CYTOKINE LEVELS FOR TREATMENTS**											
IL-10	11	269	0.371	2.731	0.006	48.837	10	0.000	79.524	2.78587	0.43505
			(0.105 to 0.637)								
IFN-γ	10	182	−0.246	−1.951	0.051	70.853	9	0.000	87.298	15.07678	0.04709
			(−0.493 to 0.001)							
IL-6	6	128	−0.478	−2.829	0.005	29.394	5	0.000	82.990	−3.82394	0.62648
			(−0.809 to −0.147)								
TNF-α	6	126	−0.617	−4.462	0.000	19.639	5	0.001	74.540	1.75891	0.79823
			(−0.887 to −0.346)								
CCL2	6	262	0.087	0.707	0.479	27.611	5	0.000	81.891	−3.99857	0.23653
			(−0.155 to 0.330)								
CXCL13	3	99	−0.192	−1.321	0.186	18.952	2	0.000	89.447	−5.83565	0.67616
			(−0.477 to 0.093)								
IL-12P40	3	52	0.988	3.717	0.000	27.669	2	0.000	92.772	11.19767	0.31339
			(0.462 to 1.514)								
IL-17	3	122	−0.410	−1.656	0.098	11.029	2	0.004	81.865	−3.76637	0.56860
			(−0.895 to 0.075)								
**D. SUMMARY OF csf CYTOKINE LEVELS FOR TREATMENTS**											
CXCL13	3	86	−0.590	−3.217	0.001	4.208	2	0.122	52.473	−5.41261	0.00827
			(−0.949 to 0.230)								

*BAFF, B-cell activating factor; df, degrees of freedom; IFN-γ, interferon; CCL, Chemokine (C-C motif) ligand; CXCL, Chemokine (C-X-C motif) ligand; IL, interleukin; TGF, transforming growth factor; TNF, tumor necrosis factor*.

### Main Associations of MS With CSF Cytokines

**Figure 3:**
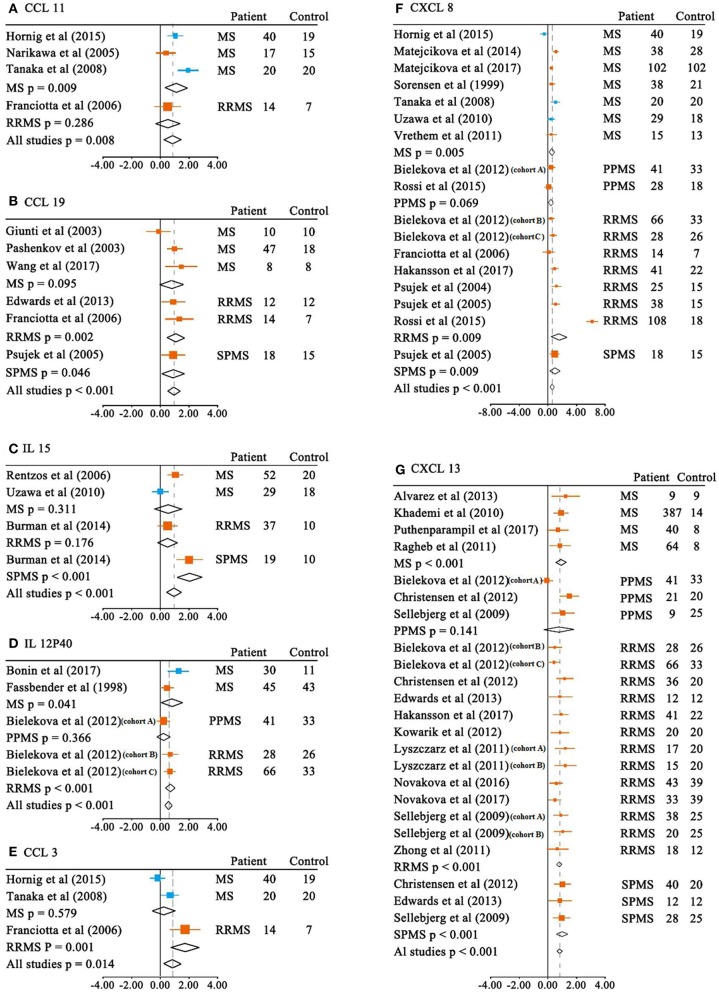
Multiple sclerosis to control SMD for CSF cytokines. CSF SMDs of CCL11 **(A)**, CCL19 **(B)**, IL-15 **(C)**, IL-12P40 **(D)**, CCL3 **(E)**, CXCL8 **(F)**, CXCL13 **(G)** between multiple sclerosis and controls. 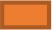 ELISA; 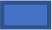 non-ELISA.

We next performed meta-analyses for the CSF cytokine differences between MS patients and the controls. Of the 26 CSF cytokines analyzed, the levels of 13 cytokines were significantly increased in MS patients when compared with the controls (Table 1B and Figure 3). For those 13 CSF cytokines significantly associated with MS, CCL21, IL-15, CCL19, CCL11, CCL3, and CXCL13 had large ESs to differentiate between MS patients and the controls (SMD 0.805–0.959), and CCL22, CXCL12, CXCL8, and IL-12p40 also had good performance to differentiate between cases and the controls (SMD 0.545–0.762), whereas those of CXCL1, IL-10, and TNF-α were moderate (SMD 0.381–0.494).

### Associations of Cytokines With Medication in MS Patients

We next explored whether cytokine levels were changed in response to drug treatments in MS patients. The random-effects meta-analysis indicated that the blood IL-12p40 and IL-10 levels were significantly increased in patients with MS after drug treatment, whereas the blood IL-6 and TNF-α levels were significantly decreased in response to drug treatment in patients (Table 1C). In addition, the other analyzed blood cytokines including CCL-2, CXCL13, IFN-γ, and IL-17 did not show significant changes after drug treatment in the MS patients.

We have also assessed the CSF cytokine changes after drug treatment in patients, and only CSF CXCL13 had enough studies to perform a meta-analysis. Our data showed that CSF CXCL13 levels were significantly decreased in the MS patients after drug treatment (Table 1D).

### Investigation of Heterogeneity

Of the 21 blood cytokines significantly associated with MS, IL-15, CXCL13, and CXCL12 did not show between-study heterogeneity, IL-17F and IL-12p70 showed small levels of heterogeneity, IL-21, IL-22, IL-2, and IL-13 showed moderate levels of between-study heterogeneity, while CCL20, IL-23, IL-12p40, IL-2R, IL-17, IL-33, IL-16, TNF-α, IL-12, CCL5, IFN-γ, and CXCL8 demonstrated high levels of heterogeneity among studies.

Of the 13 CSF cytokines significantly associated with MS, CCL22, and CXCL12 did not show between-study heterogeneity, CCL19, IL-12p40, and CXCL13 had small levels of heterogeneity among studies, CCL11, CXCL1, and IL-10 showed moderate levels of heterogeneity among studies, while the between-study heterogeneity levels were high for those of CCL21, IL-15, CCL3, CXCL8, and TNF-α.

We next tried to use sub-group and meta-regression analyses to assess the potential moderators that may explain the between-study heterogeneity, these include relevant continuous variables (age, gender, and disease severity) and categorical variables (medication status, assay type, and MS subtype). We selected cytokines that were strongly associated with MS and also had a large number of studies (at least 20 studies) to analyze the potential moderators. Therefore, we performed sub-group and meta-regression analyses on CSF CXCL13 and blood IL-17.

For CSF CXCL13, the impact of heterogeneity was slightly increased for the unmedicated group (Q_15_ = 25.323; *P* = 0.046; *I*^2^ = 40.765), and the significance of the association between elevated CXCL13 levels and MS was retained (SMD 0.753; 95% CI, 0.555–0.951; *P* < 0.001). For the medicated group, the impact of heterogeneity was reduced to zero (Q_5_ = 4.827; *P* = 0.437; *I*^2^ = 0), and a larger ES was observed (SMD 0.883; 95% CI, 0.647–1.119; *P* < 0.001). We next analyzed CSF CXCL13 levels in the RRMS patients given that most of the included studies recruited this subtype of patient, and the meta-analysis showed a highly significant association between elevated CXCL13 levels and RRMS (SMD 0.760; 95%CI, 0.603–1.917; *P* < 0.001), and no significant association between-study heterogeneity was observed (Q_12_ = 12.025; *P* = 0.444; *I*^2^ = 0.212) in this subgroup.

For blood IL-17, the impact of heterogeneity was unchanged for the ELISA method (Q_20_ = 275.637; *P* < 0.001; *I*^2^ = 92.744), and the significance of the association between elevated IL-17 levels and MS was retained (SMD 0.607; 95%CI, 0.271–0.942; *P* < 0.001). For the non-ELISA method, the impact of heterogeneity was reduced by 32% (Q_5_ = 13.270; *P* = 0.021; *I*^2^ = 62.322), and the significance of the association between elevated IL-17 levels and MS was retained (SMD 0.593; 95%CI, 0.317–0.870; *P* < 0.001). In addition, the impact of heterogeneity for the unmedicated group (Q_9_ = 71.654; *P* < 0.001; *I*^2^ = 87.440) and medicated group were unchanged (Q_11_ = 125.868; *P* = 0.352; *I*^2^ = 91.261) for blood IL-17. The significance of the association between elevated IL-17 levels and MS was retained for the unmedicated group (SMD 1.050; 95%CI, 0.544–1.556; *P* < 0.001), but not for the medicated group (SMD 0.169; 95% CI, −0.187 to 0.526; *P* = 0.352). Furthermore, the impact of heterogeneity was unchanged for the RRMS group (Q_18_ = 236.773; *P* < 0.001; *I*^2^ = 92.398), and the significance of the association between elevated IL-17 levels and MS was retained (SMD 0.552; 95% CI, 0.148–0.957; *P* = 0.007).

Meta-regression analyses revealed a significant association between age and ES for studies measuring CSF CXCL13 (regression coefficient [SE], 1.147 [0.506]; 95%CI, 0.155–2.139; *P* = 0.023), but not for blood IL-17 (regression coefficient [SE], 0.194 [1.005]; 95% CI, −1.777 to 2.164; *P* = 0.847). The analyses also showed a significant association between sex and ES for studies measuring CSF CXCL13 (regression coefficient [SE], 1.137 [0.205]; 95%CI, 0.736–1.538; *P* < 0.001), but not for blood IL-17 (regression coefficient [SE], 0.859 [0.610]; 95%CI, −0.337 to 2.055; *P* = 0.159). In addition, we found a significant association between disease severity (EDSS) and ES for studies measuring CSF CXCL13 (regression coefficient [SE], 0.755 [0.344]; 95%CI, 0.082–1.429; *P* = 0.028), but not for blood IL-17 (regression coefficient [SE], 0.329 [0.789]; 95%CI, −1.218 to 1.876; *P* = 0.677). Moreover, the quality of the studies (Newcastle-Ottawa quality assessment scale) did not significantly affect the outcome of the meta-analysis analyzing blood IL-17 (regression coefficient [SE], 1.980 [1.062]; 95%CI, −0.103 to 4.062; *P* = 0.063) and CSF CXCL13 (regression coefficient [SE], 1.255 [1.204]; 95%CI, −1.104 to 3.614; *P* = 0.297). These results indicated that age, sex, and disease severity (EDSS) had moderating effects on the outcomes of the meta-analysis.

We further performed a sensitivity analysis by removing one study at a time, and the results showed that no single study influenced the highly significant association between CSF CXCL23, blood IL-23, and MS, suggesting the robustness of the outcomes of the meta-analysis. We next examined the publication bias using the Egger' test, and most of the blood and CSF cytokines did not show significant publication bias (Table 1), suggesting that the significant associations found in our meta-analysis were not likely caused by publication bias.

## Discussion

To the best of our knowledge, our systematic review and meta-analysis is the first undertaken to study the rapidly growing literature of cytokine aberration in MS. Our study pooled data from 226 articles encompassing 13,526 MS patients and 8,428 controls and showed that 21 cytokines were significantly increased in the peripheral blood of patients. Levels of 13 CSF cytokines were also elevated in patients with MS. For those cytokines significantly associated with MS and measured in more than 10 studies, the ESs associated with CSF CXCL13 (ES = 0.805), blood IL-2R (ES = 0.820) and blood IL-23 (ES = 1.711) were the largest, and CSF CXCL8 (ES = 0.553), blood IL-2 (ES = 0.63) and IL-17 (ES = 0.622) also performed well in order to differentiate between MS patients and the controls. Moreover, we found that TNF-α, CXCL8, IL-15, IL-12p40, and CXCL13 levels were consistently elevated in the blood and CSF of patients with MS. However, we observed a non-significant increase of the inflammation marker C-reactive protein levels in MS patients, and this is likely due to the large between-study heterogeneity, making an observation of statistical significance difficult. Therefore, our study provides the most comprehensive analysis of a cytokine profile in patients with MS, clarifying inconsistent results for individual cytokines and between studies in this devastating disease.

Multiple sclerosis (MS) has long been considered a Th1-driven autoimmune disease, and the major cytokines that characterize the Th1 lineage are IFN-γ and IL-12 (Luchtman et al., 2014). Our data of heightened levels of IFN-γ and IL-12 in patients with MS supports the crucial role of Th1 cell dysfunction in the pathogenesis of MS, especially when both CSF and blood IL-12p40 levels were significantly increased in MS patients. In addition, levels of another Th1 related cytokine-IL2 were also elevated in the blood of MS patients, further supporting the role of Th1 cell induced inflammation in the development of MS. However, more recent studies have suggested that a pro-inflammatory response caused by Th17 lymphocytes is essential for the onset of MS (Luchtman et al., 2014). The cytokines define Th17 subset lymphocytes as IL-17, IL-17F, and IL21 (Sie et al., 2014), and IL-23 is important for the stabilization and expansion of the Th17 cells (Luchtman et al., 2014). Consistent with the proposed role of Th17 cells in MS pathogenesis, IL-17, IL-17F, IL21, and IL-23 levels were all elevated in the blood of MS patients as demonstrated by the meta-analysis. The involvement of Th17 cells in MS pathogenesis was further supported by an animal study showing that the experimental autoimmune encephalomyelitis mice lacking either IL-23p19 or IL-23p40 did not exhibit the MS phenotype despite the typical Th1 cell induced pro-inflammatory response in an animal model of MS (Cua et al., 2003). Due to the limited number of studies, we could not perform a meta-analysis on the CSF IL-17F, IL-21, and IL-23 levels in MS. Nevertheless, the above results suggest that both Th1 and Th17 subset lymphocytes induced inflammatory responses are crucial for the pathogenesis of MS.

The RRMS diagnosis can be made based on magnetic resonance imaging according to the revised 2010 McDonald diagnostic criteria (Polman et al., 2011), although the traditional detection of oligoclonal IgG bands in CSF is no longer required for the diagnosis of RRMS, it is still necessary to identify PPMS (Orbach et al., 2014). In addition, the field has made intense efforts to identify novel CSF biomarkers in MS due to the close proximity of CSF to the targets of autoimmune attacks, in the hope of providing information on the pathological processes, diagnosis, prognosis, and treatment response for MS. These investigations led to several potential biomarkers in CSF to be proposed, especially the biomarkers related to inflammation (Stangel et al., 2013). However, there is no consensus on the proposed biomarkers due to the poor comparability between different laboratories and insufficient validations from independent research groups. Here, we pooled the inflammatory cytokine data from the literature and standardized the mean difference of cytokines between MS patients and the controls, and identified several cytokines that were consistently elevated in the CSF of patients with MS. Of the CSF cytokines analyzed in a relatively large number of studies, 22 out of 23 of these comparisons between cases and controls for CXCL13 had an SMD above zero, and the average SMD is 0.805, suggesting the robustness of CSF CXCL13 to differentiate between MS patients and controls. Our meta-analysis also showed significantly decreased CSF CXCL13 levels after medication in patients with MS, which is consistent with the study showing the unchanged CSF CXCL13 level in long-term medicated patients with MS, when compared with the controls (Bielekova et al., 2012). Moreover, The CSF CXCL13 levels were demonstrated to be correlated with the disease course of MS (Sellebjerg et al., 2009; Brettschneider et al., 2010; Khademi et al., 2011). These results suggest that CSF CXCL13 should be used in clinical practice for the diagnosis of MS, and CSF CXCL13 also has the potential to be a biomarker for drug treatment response and disease progression of MS, although further investigations are required to validate this, due to the limited number of studies in the literature.

**Figure 4:**
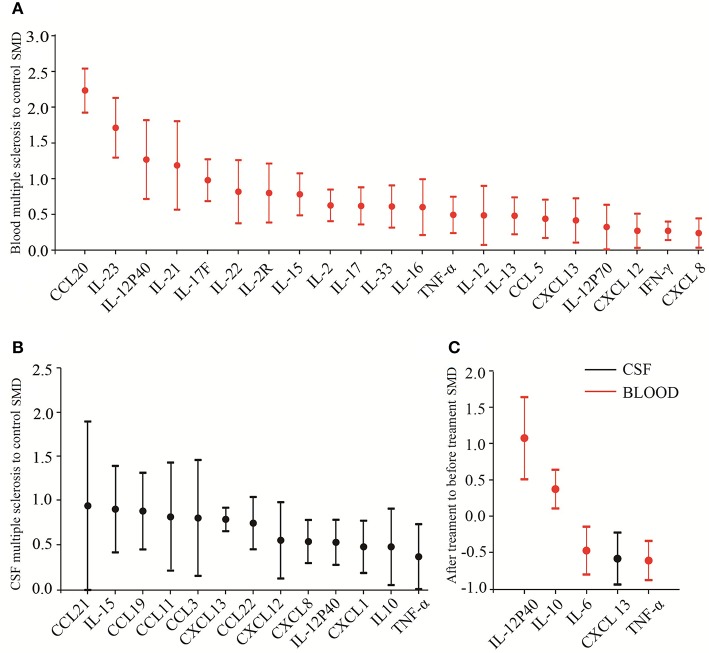
Biomarker performance rating for multiple sclerosis. Head-to-head biomarker performance in blood **(A)** and in CSF **(B)** based on multiple sclerosis to control SMD. **(C)** Biomarker performance for drug response.

In addition to the biomarker discovery in the CSF of MS patients, great efforts have been made to search for cytokine biomarkers in the blood because peripheral inflammation are also believed to be crucial for the MS pathogenesis (Hemmer et al., 2015) and blood is easily accessible. Of the blood inflammatory markers measured in at least 10 studies, IL-23 had a very large ES to differentiate between MS patients and the controls (SMD = 1.711). Ten out of 11 of these comparisons between cases and controls for IL-23 had an SMD above zero, and the only study that did not show increased blood IL-23 levels in patients with MS had a small sample size. These results suggest that blood IL-23 should be used for a biomarker to differentiate between MS patients and controls. In addition, several other blood cytokines such as IL-17F and IL-22 were also consistently elevated in MS patients with large ESs, suggesting the potential of these cytokines in the diagnosis of MS. More studies, however, are needed to validate these results. Altogether, our systematic review and meta-analysis identified a number of promising biomarkers both in the blood and CSF for the diagnosis and treatment response of MS. The head to head cytokine biomarker performance for the diagnosis and treatment response is shown in Figure 4.

The between-study heterogeneity in this meta-analysis for blood and CSF cytokines varied from zero to high. To address the between-study heterogeneity, we used subgroup and meta-regression analyses to address the potential confounders that may have moderating effects on the outcomes of the meta-analysis. Our subgroup analyses suggest that levels of between-study heterogeneity were affected by medication status for CSF CXCL13 and assay type for blood IL-17, suggesting that medication and assay type had moderating effects on the outcomes of the meta-analysis. However, another possibility of the low levels of between-study heterogeneity for some subgroups were due to the relatively small number of studies. In addition, the meta-regression analyses showed that both age, gender and disease severity were significantly correlated with ESs for studies measuring CSF CXCL13 levels. The moderating effects of age and gender on the outcomes on the meta-analysis are reasonable given that the prevalence of the disease is twice as common in women than in men (Milo and Kahana, 2010), and it has been reported that older age at MS onset had worse symptoms (Alroughani et al., 2016). Nevertheless, our findings suggest that those clinical and methodological variables need to be controlled for future studies analyzing cytokines in MS.

Although our study provided the most comprehensive analyses of the growing literature for cytokines in the blood and CSF of MS patients, the limitation of this study is that the methods used in the studies for the measurements of cytokines were research-grade but not clinically certified, therefore preventing us in making cutoff values for the diagnosis of MS. As the European Network for Biomarkers in MS recommend, future studies that measure the biomarkers for MS need to standardize CSF sampling and protocols (Teunissen et al., 2009). Our study nevertheless provides several promising biomarkers such as CSF CXCL13 and blood IL-23 to be developed as clinically certified assays for the diagnosis of MS, and this may require international collaborations. Another limitation of this study is that a limited number of studies explored the associations between disease progression and cytokine level, therefore preventing us from performing a meta-analysis for the potential prognosis of MS with inflammatory cytokines. In fact, CSF CXCL13 levels were found to be associated with the disease progression of MS (Sellebjerg et al., 2009; Brettschneider et al., 2010; Khademi et al., 2011), this highlights the need for continued investigations into the prognostic values of inflammatory cytokines for MS. Lastly, though we addressed publication bias in the meta-analysis by performing the Egger's test, we cannot exclude the impact of potential publication bias from unpublished data.

## Conclusions

The findings of the meta-analysis clarified the circulating cytokine levels in MS patients, and demonstrated that concentrations of 21 blood cytokines and 13 CSF cytokines were elevated in MS patients. These results therefore provide targets for disease modifying treatments of MS. Due to their consistency and large ESs, CSF CXCL13 and blood IL-23 should be used in clinical research and practice.

## Data Availability Statement

The raw data supporting the conclusions of this manuscript will be made available by the authors, without undue reservation, to any qualified researcher.

## Author Contributions

YC and TY conceived and designed the study. ZB, DC, LW, YZ, and TL collected the data. ZB, DC, YY, TY, and YC analyzed and interpreted the data. YC drafted the manuscript with critical revisions from all the authors.

### Conflict of Interest

The authors declare that the research was conducted in the absence of any commercial or financial relationships that could be construed as a potential conflict of interest.
