# Supplementary material for: Cerebrospinal Fluid and Blood Cytokines as Biomarkers for Multiple Sclerosis: A Systematic Review and Meta-Analysis of 226 Studies With 13,526 Multiple Sclerosis Patients
Source: Front Neurosci. 2019 Oct 4;13:1026. doi: 10.3389/fnins.2019.01026 (PMC6787166; doi:10.3389/fnins.2019.01026)
Supplement: Supplementary file 1 [file Data_Sheet_1.PDF]

Supplementary Online Content

eTable1: Characteristics of included studies measuring blood and CSF cytokine concentrations

eTable2: Newcastle-Ottawa quality assessment scale for included studies

eReference : 226 included articles in the meta-analysis [1-226]

eTable1a: Characteristics of included studies measuring blood and CSF cytokine concentrations (between-group)

Study/Year	Cytokines Measured	Country	Samples (MS/Control)	Gender(%Male) (MS/Control)	Mean Age (MS/Control)	Mean MS duration	Disease severity/ EDSS	Sample Source	Diagnosis	Assay type	Medication
Abraham et al 2017	CCL20	Israel	60/20	NA	NA	NA	NA	BLOOD	NA	ELISA	Y
Adachi et al 1990	IL-2; IL-2R	Japan	24/10	37.5/NA	31.5/NA	NA	NA	BLOOD	NA	ELISA	NA
Akcali et al 2017	IL-1β,IL-35,TNF-αIL-2 IL-10	Turkey	54/26	53.7/50	34.2/32.4	6.15	1-5.5	BLOOD	NA	ELISA	Y
Alexander et al 2010	IL-23, IL-17, IL-12p40	USA	24/16	33/37.5	24-30/24-29	1.17	2.5	BLOOD	McDonald criteria	ELISA	N
Alsahebfosoul et al 2017	IL-33	Iran	44/44	14/14	NA	NA	NA	BLOOD	McDonald criteria	ELISA	Y/N
Altıokka-Uzun et al 2015	TNF-α,IL-4,IFN-γ,IL-12,IL-10,IL-17	Turkey	13/20	NA	NA	NA	NA	BLOOD	NA	ELISA	Y
Alvarez et al 2013	CXCL13	USA	9/9	11.1/22.2	36/39.3	NA	3	CSF	2005 revised McDonald criteria	ELISA	Y/N
Arababadi et al 2010	IL-17A, IFN-γ, IL-12, IL-10	Iran	70/100	25/21	36.3/35.1	NA	NA	BLOOD	NA	ELISA	Y
Babaloo et al 2015	IL-17A,IL-17F	Iran	35/35	43/49	28.8/33	NA	NA	BLOOD	NA	ELISA	N
Bahner et al 2002	IL-12P40,IL-12P70	Germany	18/18	67/67	47/47	NA	4.5	BLOOD	modified Poser criteria	ELISA	N
Balasa et al 2015	IL-17,IL-10,TGF-beta-1	Romania	32/32	25/25	35.44/34.9	6.17	1.67	BLOOD	the McDonald diagnostic criteria 2010	ELISA	Y/N
Bansil et al 1991	IL-2R	USA	43/34	39.1/67.8	44/37	NA	10.92	BLOOD	NA	ELISA	Y/N
Bartosik-Psujek et al 2004	CCL2, CCL5	Poland	30/15	43/33	31.1/30.9	3.5	3.3	BLOOD	McDonald criteria	ELISA	N

Bartosik-Psujek et al 2004	IL-8	Poland	25/15	28/40	34.2/37.6	6.4	3	CSF/BLOOD	McDonald criteria	ELISA	N
Bartosik-Psujek et al 2005	CCL2,CCL 5,IL-8	Poland	56/15	28.39/33	33.54/30.9	8.56	3.78	CSF/BLOOD	Poser criteria	ELISA	N
Bielekova et al 2012(COHORT 1)	IL-12p40,CXCL13, IL-8	America	31/26	19.6/23	39.55/	NA	1.98	CSF	McDonald criteria	ELISA	N
Bielekova et al 2012(COHORT 2)	IL-12p40,CXCL13, IL-8	America	107/33	54.41/18	44.52/48.1	NA	3.04	CSF	McDonald criteria	ELISA	N
Bonin et al 2017	IL-17,IL-12P40	Italy	30/11	23.4/73	40.22/61.6	NA	1.9	CSF	the 2010 McDonald Revised Criteria	Bio-Plex cytokine assay panels	Y
Burman et al 2014	IL-5,IL-6,IL-9,IL-10,IL-12p70,IL-15,IL-27,IL-28A,CCL2, CCL4,CCL5,CCL7,CCL20,CCL22, CX3CL1, CXCL1,CXCL10,FGF-2,FLT-3L,PDGF-AA, PDGF-BB,sCD40L,TGF-α,TNF-α,VEGF	Sweden	56/10	33.95/30	46.2/33.5	4.75	11.59	CSF	The revised McDonald’s criteria	ELISA	N
Buttmann et al 2004	CXCL10,CCL2	Germany	62/15	25/27	41.6/40	9.08	2.1	BLOOD	McDonald criteria	ELISA	N

Cala et al 2016	IFNγ,IL-12p70,TNFβ,IL-2,IL-4,IL-33,IL-5,IL-10,IL-13, IL-25,IL-6,IL-1β,IL-23,IL-17A,IL-17F,IL-21,IL-22, TNF-α,GM-CSF,CCL20,IL-9,IL-15,IL-27,IL-28A,IL-31	USA	40/11	29/55	15.28/13.76	2.01	NA	BLOOD	NA	ELISA	Y
Campbell et al 2010	IL-8	UK	10/10	NA	NA	NA	NA	BLOOD	NA	cytometric bead array (CBA)	N
Castellano et al 2008	IL-6, TNF-α, IFN-γ	Florida	11/11	27/27	40/40	NA	0-5.5	BLOOD	Poser criteria	multiplex immunoassay	N
Chalon et al 1992	IL-2R	Belgium	59/19	NA	40.98/NA	NA	NA	BLOOD	NA	ELISA	Y
Chen et al 2012	IL-17,IL-18,IL-23	China	39/39	36/36	49.4/49.4	6.8	4.7	BLOOD	McDonald (2001) criteria	FlowCytomix Assay kits	N
Chen et al 2012	IL-6	China	39/39	36/36	49.4/49.4	6.8	4.7	BLOOD	McDonald (2001) criteria	FlowCytomix Assay kits	N
Christensen et al 2012	CXCL13	Sweden	97/20	46.52/55	44.04/53	10.93	4.25	CSF	the McDonald criteria	ELISA	N
Christophi et al 2011	IFN-γ,IL-4,IL-13, TNF-α IL-6	USA	52/32	30.62/31	40.81/41	5.71	2.48	BLOOD	NA	ELISA	Y/N
Christophi et al 2012	IL-33,IL-6,IFN-γ, IL-13	USA	52/39	30.62/31	40.81/41	5.71	2.48	BLOOD	NA	ELISA	Y/N

Comini-Frota et al 2011	CXCL10,CCL2,CCL4,CCL5,CXCL9	Brazil	28/28	25.18/32	34.07/33	5	1.22	BLOOD	the McDonald criteria	ELISA	N
Damasceno et al 2016	IL-10, IL-6, TNF-α and IFN-γ	Brazil	21/8	28.6/37.5	27.67/29.7	4.52	2.21	BLOOD	NA	ELISA	NA
De Flon et al 2018	CXCL10,IL-12P40	Sweden	70/55	32/49	41.3/37.6	9.6	1.5	CSF	NA	multiplex electrochemiluminescence assay	Y
Drulovic et al 1998	TNF-α, IL-12, TGF-β1	Yugoslavia	21/13	NA	NA	6.6	4	BLOOD	Poser et al. 1983	ELISA	NA
Drulovic et al 1997	IL-12,INF-γ	Yugoslavia	70/52	23.9/NA	34/NA	4	4	CSF	Poser et al. 1983	ELISA	NA
Duddy et al 1999	IL-10,IL-6,IL-12, TNF-α, IL-1β	UK	18/18	39/NA	35/NA	10	4	BLOOD	Poser et al. 1983	ELISA	NA
Edwards et al 2013	CXCL12,CXCL13, CCL19,CCL21	America	24/12	25.5/37.5	46/38	NA	NA	BLOOD/CSF	NA	ELISA	N
Emamgholipour et al 2013	TNF-α,IL-1β, hs-CRP	Iran	191/200	32.5/33	35.16/36.11	NA	NA	BLOOD	the McDonald criteria	ELISA	NA
Esendagli et al 2013	IL-17,IL-23,IL-26	Turkey	21/8	60/25	31.93/31.12	1.5	1.53	BLOOD	the McDonald criteria	ELISA	Y
Fan et al 2015	IL-21	China	25/14	32/36	40/38.5	2	2	BLOOD	the 2010 McDonald's diagnostic	ELISA	N
Farhadi et al 2014	IL-4, IFN-γ, and TNF-α	Iran	30/30	20/27	28.2/28.64	NA	NA	BLOOD	NA	ELISA	NA
Farrokhi et al 2015	IL-37	Iran	122/49	32/27	37.96/35.26	4.73	2.59	BLOOD	MacDonald criteria	ELISA	NA
Farrokhi et al 2015	TNF-α	Iran	17/17	82/82	34.35/31.82	5.88	2.52	BLOOD	MacDonald criteria	ELISA	N

Farrokhi et al 2017	IL-16	Iran	250/400	37.6/35.5	34.67/34.14	4.9	NA	BLOOD	MacDonald criteria	ELISA	N
Fassbender et al 1998	IL12p40,IL 12p70, TNF-α,IL1β,MBP	Germany	45/43	42.2/81	33/38	NA	NA	BLOOD/CSF	Poser et al. 1983	ELISA	NA
Fjeldstad et al 2011	CRP	USA	14/13	29/54	47/44	8	2.5	BLOOD	NA	ELISA	NA
Franciotta et al 2006	XCL1, CXCL1, CXCL7, CXCL8, CXCL10, CXCL11, CXCL12, CCL1, CCL2 CCL3,CCL 4,CCL5,CC L11,CCL17, CCL19,CC L20,CCL21, CCL22	Italy	14/7	36/57	30/35	NA	NA	BLOOD/CSF	NA	ELISA	N
Franciotta et al 1989	TNF-α	Italy	50/10	46/NA	37.5/NA	6.3	NA	BLOOD/CSF	NA	ELISA	N
Galimberti et al 2008	CCL22	Italy	56/15	32/0	38.3/38.18	NA	NA	CSF	MacDonald criteria	ELISA	NA
Gallo et al 1988	IL-2	Italy	30/33	30/NA	34.5/27	NA	NA	BLOOD/CSF	NA	ELISA	NA
Gallo et al 1989	IL-2, TNF-γ, sIL-2R	Italy	50/30	32/40	NA	NA	NA	BLOOD/CSF	NA	ELISA	NA
George et al 2012	IL-33,IL-6,IFN-γ, IL-13	USA	32/32	31/31	41/41	5.9	2.5	BLOOD	NA	ELISA	N
Giunti et al 2003	CXCL10,C XCL12, CCL19	Italy	21/10	29/NA	36.6/NA	NA	NA	CSF	McDonald criteria	ELISA	NA

Glasnovic et al 2014	CRP,TNFα, IL-1β	Croatia	27/30	NA	37.7/43.6	NA	3	BLOOD/CSF	the revised McDonald criteria	ELISA	NA
Guerrero-Garcia et al 2016	IFN-γ, IL-10, IL-17A	Mexico	82/25	39/40	33/NA	7.9	2.5	BLOOD	revised McDonald criteria	ELISA	Y
Haas et al 2017	IL-17A	Germany	77/20	27/35	35.43/31.4	7.68	2.75	BLOOD/CSF	the revised McDonald criteria	ELISA	Y/N
Hagman et al 2011	CCL2,CCL3,CCL4,CXCL10,IL-10,TNF–α,IL-6,IL–12p70 IFN-γ, IL-2	Finland	72/21	33/38	46/42	5.8	3	BLOOD	The revised McDonald’s criteria	ELISA	Y
Hakansson et al 2017	CXCL1,CXCL8, CXCL10,CXCL13,CXCL20,CCL22	Sweden	41/22	22/23	31/32	NA	NA	BLOOD/CSF	2010 McDonald's criteria	ELISA	N
Hashemi et al 2006	IL-10	USA	125/23	25.15/30	45.98/41.65	4.9	2.04	BLOOD	McDonald criteria	ELISA	Y
Hedegaard et al 2010	BAFF	Denmark	49/12	35/NA	32.65/NA	2.42	1.69	BLOOD	NA	ELISA	N
Heesen et al 1999	IL-1ra	Germany	66/10	28.6/NA	NA	NA	NA	BLOOD	Poser et al	ELISA	NA
Hietaharju et al 2010	IL-6	Finnish	12/12	NA	50.67/50.67	14.33	4.13	BLOOD/CSF	NA	ELISA	NA
Hohnoki et al 1998	INF-γ,TNF-α, IL-4,IL-10	Japan	21/12	28.65/50	36.6/28.3	NA	NA	BLOOD	Poser et al. 1983	ELISA	NA
HOLLIFIELD et al 2003	TNF-α IL-1β, IFN-γ, TGF-β1	UK	15/10	54/60	39.67/39	4.59	3.12	BLOOD	McDonald criteria	ELISA	N

Hornig et al 2015	IL1ra,IL1α,IL1β,IL-2,IL-4,IL-5,IL-6,IL-7,CXCL8,IL-10,IL12p40,IL12p70,IL-13,IL-15,IL-17A,IL-17F,IFNα2,IFNβ,IFNγ, TNFα,TNFβ,CD40L,sFasL,TRAIL,CCL2,CCL3, CCL4,CCL5,CCL7,CCL11,CXCL1,CXCL5,CXCL9,CXCL10,TGFα,TGFβ,SCF(SF),CSF1(MCSF),CSF2(GMCSF)CSF3 (GCSF),PDGFBB,βNGF,FGFb,HGF,VEGFA,LIF,Resistin, Leptin,Serpin E1 (PAI1),sICAM1 (CD54),VCAM1 (CD106)	USA	40/19	30/31.6	44.2/50.5	NA	NA	CSF	NA	a magnetic bead-based 51-plex immunoassay	NA
Huber et al 2014	IFNγ/CXCL1/CCL11	Canada	38/25	57.89/57.7	51.43/53.8	17.79	4.41	BLOOD	the revised McDonald Diagnostic Criteria	multiplex magnetic bead-basedarrays	NA

Jafarzadeh et al 2013	CCL22	Iran	135/135	21.5/25.9	35.72/36.5	NA	NA	BLOOD	the McDonald criteria	ELISA	Y/N
Jafarzadeh et al 2014	IL-35	Iran	140/140	32.9/33.6	34.98/36.07	NA	NA	BLOOD	the McDonald criteria	ELISA	Y/N
Jafarzadeh et al 2014	CCL20	Iran	135/135	21.5/25.9	35.72/36.5	NA	NA	BLOOD	the McDonald criteria	ELISA	Y/N
Jafarzadeh et al 2016	IL-33	Iran	160/160	31.4/32.3	34.83/36	NA	NA	BLOOD/CSF	The revised McDonald's criteria	ELISA	Y/N
Jensen et al 2004	TNF,IL-12p40	Denmark	10/10	40/40	41/32	7	3.5	BLOOD	NA	ELISA	N
JI et al 2016	hs-CRP, TNF-a, IL-10	China	36/10	78/60	34.6/36.1	NA	NA	BLOOD/CSF	McDonald criteria	ELISA	NA
Kalinowska-Lyszczarz et al 2011	CXCL13CCL17, CCL20,IL-17	Poland	21/20	25/15	36.21/NA	2.4	2.69	BLOOD/CSF	revised McDonald criteria	ELISA	N
KALLAUR et al 2013	IL-1β,IL-6,TNF-α,IL-12,IFN-γ,IL-17, IL-4, IL-10	Brazil	169/132	27.2/21.2	42/38.5	7	0-10	BLOOD	the revised McDonald Criteria	ELISA	Y
KALLAUR et al 2016	IL-1β, IL-6, TNF-α, IFN-γ, IL-17,IL-4,IL-10, IL-12	Brazil	158/40	37.5/NA	37.8/NA	5.58	1.51	BLOOD	the revised McDonald Criteria	ELISA	NA
KALLAUR et al 2016	IL-6,IL-4,IL-10, TNF-α,IFN-γ,IL-17	Brazil	212/249	29/29	42.7/36.8	7.35	NA	BLOOD	the revised McDonald Criteria	ELISA	Y
Kannel et al 2015	BAFF	Estonia	170/49	39/18	39.2/37.2	11.3	NA	BLOOD	the McDonald criteria	ELISA	NA
Khademi et al 2010	CXCL13	Sweden	387/14	32.29/21	41.21/29.7	7.13	2.78	CSF	the McDonald criteria	ELISA	Y/N

Khaiboullina et al 2015	IL-1α,IL1b,IL-1ra,IL-2Ra,IL-2,IL-3,IL-4,IL-5,IL-7,IL-8,IL-9,IL-10, IL-12p40,IL-12(p70),IL-13,IL-15,IL-16,IL-17A,IL-17F,IL-18,IL-21,IL-22,IL-23,IL-25,IL-31,IL-33,CCL2,CCL3,CCL4,CCL5,CCL7CCL27,CXCL9,CXCL10,CXCL12,INFγ,TNFα, TNFβ	Russia	42/20	41.6/NA	4.8/NA	12.4	3.5	BLOOD	the McDonald 2010 criteria	Bio-Plex multiplex magnetic bead-based antibody detection kits	Y/N
Kittur et al 1990	IL-2R	US	32/35	NA	NA	NA	NA	BLOOD	NA	ELISA	NA
Kostic et al 2014	IL-17A	Serbia	39/40	40/73	33/63	1	3.5	CSF	2010 revised McDonald criteria	ELISA	N
Kowarik et al 2012	CXCL12CXCL13 CCL19,CCL21	Germany	20/20	45/50	38/32	NA	NA	CSF	NA	ELISA	N
Kreft et al 2012	IL-7	Netherlands	40/40	25/42	32.94/41	7.58	NA	BLOOD	the McDonald criteria	ELISA	NA
Kurne et al 2010	BAFF,sTRAIL	Turkey	35/19	40/NA	25.4/NA	6.05	2.4	BLOOD	McDonald criteria	ELISA	N
Kurtuncu et al 2012	IL-17,IL-23,IL-10,IL-	Turkey	63/30	32/30	35.2/37.5	3.9	1.9	BLOOD	the McDonald criteria	ELISA	N

	4,IFN-γ,IL-9TGFβ											
Lebrun et al 2016	IL-17	USA	1257/80	26/47	28.5/33.2	NA	NA	BLOOD/CSF	NA	ELISA	Y/N
Losy et al 2002	IL-12,TGF-β1	Poland	20/22	20/20	32.6/32.6	4.4	2.6	BLOOD	Poser criteria	ELISA	N
Losy et al 2002	IL-10, IL-12	Poland	31/30	35/35	34/34	5	2.7	BLOOD	Poser criteria	ELISA	N
Lund et al 2004	CXCL8	US	104/31	35.5/32	46.27/38	9.06	4.62	BLOOD	Poser criteria	cytometric bead array (CBA)	Y/N
Lundstrom et al 2014	IL-7	Sweden	205/6	NA	NA	NA	NA	BLOOD	NA	ELISA	Y
Mahad et al 2002	CCL2, CXCL10CXCL19	UK	43/44	35/65	38/55	NA	NA	CSF	Poser criteria	ELISA	N
Maimone et al 1991	TNF-α,IL-1β, IL-6	USA	34/43	20.6/46.5	40.8/49.9	NA	NA	BLOOD/CSF	NA	ELISA	N
Malekzadeh et al 2017	IL-1β, IL-6, IL-8 TNF-a	Netherlands.	57/203	42/58	45.2/54	NA	3.99	BLOOD/CSF	NA	MSD platform	NA
Malmestrom et al 2006	IL-4,IL-6,IFN-γ,TNF-α	Sweden	63/44	38/71	39.7/36	14.41	4.22	BLOOD/CSF	the McDonald criteria	Bio-Plex human cytokine assays	Y
Martinez-Caceres et al 2002	CCL5, CXCL10	Spain	68/26	31/42	39.5/36	10.8	4.9	CSF	Poser criteria	ELISA	N
Martins et al 2011	IFN-γ IL-1β IL-2 IL-4 IL-5 IL-6 IL-8 IL-10 IL-12 IL-13 TNFα IL-2r	America	833/117	23.2/28.2	47/47	11.2	NA	BLOOD	the McDonald criteria	The Luminex Multi-Analyte Profiling system	Y/N
Matejcikova et al 2014	IL-6, IL-8, IL-10	Czech	38/28	34/28.6	35.8/39.7	NA	NA	BLOOD/CSF	McDonald’s revised	ELISA	N

									diagnostic criteria		
Matejcikova et al 2017	IL-8	Czech	102/102	31.4/22.5	37.5/40	NA	NA	BLOOD/CSF	McDonald's revised diagnostic criteria	ELISA	N
Matsushita et al 2013	IL-17A,CXCL 8,<br>IL-6,<br>CXCL10<br>CCL4	Japan.	35/18	43.22/67	39.77/46.3	9.17	4.45	CSF	the McDonald criteria	ELISA	Y/N
mellergard et al 2010	IL-1β, IL-2,IL-4, IL-5, IL-6, IL-8, IL-10, TNF-α,<br>IFN-γ, GM-CSFCXCL9 CXCL10CXCL11CCL17, CCL22	Sweden	31/15	42/NA	35.77/NA	8.98	3.9	BLOOD	NA	multiplex bead assay	Y
Michalopoulou et al 2004	sIL-6R	Greece	58/13	NA	NA	NA	NA	CSF	Poser criteria	ELISA	NA
Mikulkova et al 2011	IFN-γ TNF-α<br>IL-17A IL-2 IL-6<br>IL-8 IL-12p70 IL-4<br>IL-10<br>TGFβ1	Czech	58/40	18.9/70	32.72/28	NA	NA	BLOOD	McDonald Diagnostic Criteria	bead-based analyte detection assay	N
Miljkovic et al 2002	IL-6	Yugoslavia	50/23	22/NA	35.1/NA	4.6	3.5	CSF	Poser criteria	ELISA	NA
Mirandola et al 2009	IL-12	Brazil	106/30	28.5/37	37.4/34	13.24	5.04	BLOOD	Poser criteria	ELISA	Y/N
Mori et al 2016	CCL5	Italy	92/44	33/27	34.8/44.6	NA	NA	CSF	NA	Bio-Plex Multiplex Cytokine Assay	N

Morsaljahan et al 2017	IL-32	Iran	132/171	18.2/25.1	32.3/31.05	4.4	1.46	BLOOD	McDonald criteria	ELISA	Y
Mouzaki et al 2015	IFN-γ,TNF-α, TGF-β1,IL-2,IL-4, IL-6,IL-10,IL-17	Greece	46/40	41/42.5	34.04/37.8	NA	2.5	BLOOD/CSF	2005 revised McDonald criteria	ELISA	NA
Muls et al 2017	IL-22	America	27/14	22.6/28.6	38.13/34.9	8.6	2.54	BLOOD/CSF	revised McDonald's Criteria	ELISA	NA
Musabak et al 2010	TNF-α IL-12p70 IL-13	Turkey	57/34	46/44	36.6/37.1	8.5	NA	BLOOD	2005 McDonald revised criteria	ELISA	Y
Naderi et al 2016	IL-27	Iran	40/40	17.5/17.5	NA	NA	NA	BLOOD	the McDonald Criteria	ELISA	NA
Narikawa et al 2005	CXCL10 CCL17 CCL11 CCL2	Japan	17/15	18/27	24/46	4	NA	BLOOD/CSF	McDonald criteria	ELISA	NA
Nicolleti et al 1996	IL-12	Italy	15/30	60/60	48.6/48.6	15	5.4	BLOOD	Poser et al (1983 Protocols)	ELISA	N
NICOLETTI et al 1998	TGF-β1	Italy	55/29	40/51.7	41/41	5.1	NA	BLOOD	Poser et al. 1983	ELISA	Y
Nicolleti et al 2000	IL-1β,IL-2,IL-4, IL-6,IL-10,INF-γTNF-α	Italy	14/14	NA/42.8	28/28	6.5	1.9	BLOOD	Poser criteria	ELISA	N
Niedziela et al 2016	IL-1/IL-1F2/CRP	Poland	63/10	28.56/39.3	42.19/40	6.3	2.63	BLOOD	2010 revised McDonald criteria	ELISA	Y
Nischwitz et al 2014	IL-16	Germany	16/17	31/24	32.6/33.1	NA	1.7	BLOOD	NA	ELISA	Y
Novakova et al 2016	CXCL13CCL2	Sweden	43/39	37.21/64.1	39.65/33.59	NA	2.5	CSF	revised MacDonald criteria	ELISA	N
Novakova et al 2017	CXCL13CCL2	Sweden	59/39	39/64	37/34	NA	2.5	CSF	McDonald's criteria	ELISA	Y

Obradovic et al 2012	TNF-α, IL-4	Serbia	60/20	40/40	43.5/43.5	5.6	3.8	BLOOD/CSF	Poser criteria	ELISA	N
Oliveira et al 2016	IL-6, CRP	Brazil	150/249	26/28.92	41.42/36.8	NA	2.74	BLOOD	revised MacDnald criteria	ELISA	NA
Oliveira et al 2017	CRP	Brazil	258/249	29.9/28.9	43.2/36.7	NA	NA	BLOOD	McDonald criteria	a turbidimetric assay	Y/N
Orhan et al 2016	IL-18	Turkey	112/123	27.4/51.5	36.4/32.8	NA	1	BLOOD	revised MacDonald criteria	ELISA	NA
Padberg et al 1999	sIL-6R,IL-6R,	Germany	61/22	27.8/50	53.87/48.7	NA	NA	BLOOD/CSF	Poser et al. 1983	ELISA	NA
Paroni et al 2017	IFN-γ, IL-17, IL-22,CCL10/CCL21	America	55/25	NA	NA	NA	NA	BLOOD/CSF	the McDonald criteria	ELISA	NA
Pashenkov et al 2003	CCL12,CCL19,CCL21	Sweden	47/18	21/22	42/47	2	NA	BLOOD/CSF	the McDonald、Poser criteria	ELISA	N
Perriard et al 2015	IL-22	Switzerland	63/13	30/46.7	41.89/34	8.92	2.73	BLOOD	revised McDonald criteria	ELISA	NA
Piazza et al 2010	BAFF	Italy	30/22	33/14	37.1/38.4	NA	NA	BLOOD/CSF	the criteria of McDonald	ELISA	N
POLACHINI et al 2014	TNFα, IFN-γ, IL-1, IL-6, IL-10 CRP	Brazil	29/29	34/31	39.5/36.4	9.65	NA	BLOOD	the McDonald criteria	ELISA	NA
Puthenparampil et al 2017	CXCL13 IL-21	Italy	40/8	35/23.5	37.8/43.2	0.5	NA	CSF	NA	ELISA	N
Puthenparampil et al 2016	BAFF	Italy	40/18	35/28	36.6/41.9	0.4	NA	BLOOD/CSF	NA	immune-enzymatic assay	N
Ragheb et al 2011	BAFF, CXCL13	USA	62/8	22/13	43.9/66	NA	2.76	CSF	the McDonald/Polman criteria	ELISA	N

Ramos-Cejudo et al 2011	IL-1α IL-1β IL-2 IL-4 IL-5 IL-6 IL-8 IL-10 IL-12p70 IL-13 IL-17 IFN-γ TNF-α	Spain	22/11	25/46	35.53/NA	9.95	3.69	BLOOD	the McDonald criteria	flow cytometry	Y
Reale et al 2015	IL-1β, IL-17	Italy	15/15	20/13.3	38/40	5	2.5	BLOOD	NA	ELISA	N
Rentzos et al 1996	TNF-α	Greece	30/20	40/60	34/55	NA	NA	BLOOD/CSF	Poser et al (1983 Protocols)	ELISA	NA
Rentzos et al 2006	IL-15	Greece	52/20	48/NA	35/NA	7	3	BLOOD/CSF	the criteria of McDonald	ELISA	NA
Rentzos et al 2010	IL-15	Greece	52/21	36.52/38	35.06/37	7.12	6.18	BLOOD	the criteria of McDonald	ELISA	N
Rollnik et al 1997	TGF-β1	Gernamy	64/20	37.5/NA	35.93/NA	4.55	2.72	BLOOD/CSF	NA	ELISA	NA
Rossi et al 2011	IL-4,IL-5,IL-10, IL-13	Italy	52/29	29/31	34.5/35.3	3.6	NA	CSF	McDonald criteria	Bio-Plex Multiplex Cytokine Assay	NA
Rossi et al 2013	TNF-a,IL-1β, IFN-γ	Italy	101/33	37.4/27	34.55/37.5	4.33	2.15	CSF	NA	Bio-Plex Multiplex Cytokine Assay	NA
Rossi et al 2015	IL-8	Italy	136/18	34.26/39	33.88/32.9	NA	2	CSF	NA	ELISA	NA
Rudick et al 1990	sIL-2R	Italy	34/15	29.4/29.4	36.3/36.3	NA	NA	BLOOD	NA	ELISA	N
Huber et al 1990	sIL-2R,IL-2	Italy	8/50	25/NA	34.8/NA	7.8	NA	BLOOD	NA	ELISA	N
Ruocco et al 2015	IL-9	Italy	107/70	39/39	32/31.6	4.8	1.6	CSF	2010 revisions McDonald criteria	ELISA	Y
Sadowska-Bartosz et al 2013	CRP	Poland	57/12	25.5/33	42.67/37.3	5.88	3.03	BLOOD	the McDonald criteria	dry chemistry immunological method	Y/N

Salama et al 2003	TNF-α, IL-2R, IL-10 IL-12	USA	55/27	32.73/30	39.42/33.7	8.7	5.94	BLOOD	NA	ELISA	N
Salehi et al 2016	TGFβ,IL-6,IL-23	Iran	42/15	12/33	34/28.6	6.98	3.51	BLOOD	the revised McDonald's criteria	ELISA	N
Salmaggi et al 1995	IL-10	Italy	41/17	39/29.4	37.5/35.6	9.2	NA	BLOOD	Criteria of Mcdonald and Halliday	ELISA	NA
Saruhan-Direskeneli et al 2003	CXCL10 CXCL8 CCL2 IL-12 IL-10 IL-17 CXCL10 CXCL8 CCL2 IL-12 IL-10 IL-17	Turkey	25/14	32/29	36.1/46.7	NA	NA	BLOOD/CSF	NA	ELISA	Y/N
Sayad et al 2014	IL-2	Iran	100/100	NA	27/29	NA	NA	BLOOD	the McDonald criteria	ELISA	NA
Sayad et al 2014	IL-2	Iran	260/450	37/39	34.03/32.53	6.23	3.45	BLOOD	the McDonald criteria	ELISA	NA
scalabrino et al 2010	TNF-α	Italy	28/22	27.85/35.42	43.21/45.17	NA	NA	CSF	NA	a highly sensitive solid-phase enzyme immunometric assay	NA
Sellebjerg et al 2009	CXCL13	Denmark	95/25	44/28	42.55/34	NA	NA	CSF	NA	ELISA	Y/N
Sellner et al 2008	IL-1β IL-2 IL-6 IL-12p70 TNF-α IFN-γ IL-4 IL-5 IL-10	Switzerland	24/8	NA	31.6/34	3.5	2	BLOOD	the criteria of McDonald	The human LINCOplex High SensitivityCytokine kit	N
Sexton et al 2014	CCL2, IL-17	USA	10/11	30/45	32.8/37.6	NA	NA	BLOOD	NA	multiplex microbead-based	Y/N

										immunoassay kit	
Shajarian et al 2014	IL-23	Iran	40/40	17.5/30	30.89/32.75	NA	NA	BLOOD	the McDonald criteria	ELISA	Y/N
Sharief et al 1991	TNFα	UK	38/28	36.8/NA	31.6/NA	6.4	5.1	BLOOD/CSF	NA	ELISA	NO
Shu et al 2017	CRP	China	87/80	36.8/41.2	34.2/36.9	1	2	BLOOD	McDonald's criteria	Hitachi High Technologies	NA
Sorensen et al 1999	IP-10 Mig GRO-a IL-8 RANTES MCP-1 MIP-1a	USA	38/21	44.7/42.9	34/48	NA	NA	CSF	Poser et al. 1983	ELISA	NA
Sorensen et al 2004	CCL2	USA	15/14	20/28.57	38/54	NA	2.08	CSF	Poser criteria	ELISA	N
Stojkovic et al 2014	CXCL16	Serbia	459/303	38/51	38/39.6	10.2	4.2	BLOOD	NA	ELISA	NA
Su et al 2014	IL-7	China	26/26	19/23	NA	5.4	4.6	BLOOD	2010 McDonald's criteria	ELISA	NA
Sumita et al 2012	BAFF, IL-6	Japan	24/34	54/50	39.8/55.9	NA	NA	CSF	NA	ELISA	
Szczucinski et al 2010	CCL5, CXCL10, CXCL11	Poland	47/25	75/88	37.16/33.4	2.57	2.45	BLOOD/CSF	Mc Donald criteria	ELISA	
Tanaka et al 2008	IL-6 CXCL8 IL-10 IL-17 IFN-γ CXCL10 TNF-α CCL11 CCL3	Japan	20/20	30/65	45.8/47	NA	NA	CSF	Poser criteria	Multiplexed fluorescent bead-based immunoassay	
Tang et al 2015	IL-27,IFN-γ,IL-17	China	45/25	44/44	NA	NA	NA	BLOOD	revised MacDonald criteria	ELISA	
Tao et al 2014	IL-17A IL-17F IFN-γ IL-4	China	23/22	NA	NA	NA	NA	BLOOD/CSF	Mc Donald criteria	ELISA	

Tawfik et al 2016	IL-17 IL-10	Egypt	30/15	50/46.67	30.2/30.4	5	3	BLOOD	revised MacDonald criteria	ELISA
Tejera-Alhambra et al 2015	HGF CCL11 CCL2 CCL5 EGF CCL4	MEXICO	129/53	34/45	42/37	11	2.5	BLOOD	McDonald’s criteria	multiplex array
Tiumentseva et al 2016	IL-18	Russia	51/28	33/18	33.06/35.11	NA	NA	BLOOD	McDonald’s criteria	ELISA
Tmioka et al 1992	IL-2	Japan	46/35	25/NA	32.5/NA	NA	NA	BLOOD/CSF	NA	ELISA
Tong et al 2018	CCL13 CCL2 CCL8 CCL7 CCL11 CCL24 CCL26 TNF-α IL-1β	China	47/30	28/27	34.96/33.9	3.36	2.64	BLOOD	2015 revised international criteria and the 2010 McDonald’s diagnostic criteria	MILLIPLEX® map human High Sensitivity Cytokine/Chemokine Panels
Trenova et al 2014	TNF-a, IFN-γ, IL-4, IL-10	Bulgaria	35/35	0/0	34.8/30.45	5.66	3.12	BLOOD	2000 McDonald's criteria	ELISA
Trenova et al 2017	TNF-α, IL-17A	Bulgaria	220/99	25.5/28.3	39.05/39.28	11.43	1.9	BLOOD	the McDonald criteria (2010)	ELISA
Trotter et al 1989	IL-2	USA	40/12	30/NA	40.03/NA	NA	6.16	BLOOD	NA	ELISA
Trotter et al 1990	IL-2	USA	60/61	NA	NA	NA	NA	BLOOD	NA	ELISA
Trotter et al 1991	IL-1α IL-1β IL-2 TNF-α sIL-2R IL-6	USA	126/81	NA	NA	NA	NA	BLOOD	NA	ELISA
Tsukada et al 1991	IL-2R	Japan	40/16	40/43.7	38/40.5	NA	NA	BLOOD	NA	ELISA
Tsukada et al 1991	TNF-α	Japan	31/18	35.5/NA	38/40.5	NA	NA	BLOOD/CSF	NA	ELISA

Tumani et al 2011	IFN-γ IL-2, IL-4, IL-10	Germany	19/29	32/28	34/34	1.3	2	BLOOD	the revised McDonald criteria	electrochemiluminescence detection multiplex technology
Uzawa et al 2010	IL-1ra IL-2 ,IL-4 IL-5 ,IL-6 IL-7 ,IL-8 IL-9 IL-10 IL-12(p70) IL-13 IL-15 IL-17 IFN-γ TNF-α IL-1β	Japan	29/18	24/NA	30.6/NA	3.75	3.5	CSF	the criteria of McDonald	multiplexed fluorescent magnetic bead-based immunoassay
Vrethem et al 2011	IL-1β IL-6 CXCL8 IL-10 TNF	Sweden	25/13	40/62	46.4/52	11	3.2	BLOOD/CSF	the McDonald criteria	ELISA
Wang et al 2012	IL-6, SIL-6R	China	18/14	39/43	35.5/50	4	2.75	CSF	2010 McDonald's criteria	ELISA
Wang et al 2013	IL-6, IL-17	China	18/14	39/43	35.5/50	4	2.75/	CSF	2010 McDonald's criteria	ELISA
Wang et al 2017	CXCL13	China	20/22	30/27	31.8/33.59	3.63	NA	BLOOD	the 2010 McDonald's diagnostic criteria	ELISA
Wang et al 2017	IL-17A IL-6 CCL19 IL-19	China	8/8	25/25	45/40	4.8	2.86	BLOOD/CSF	the McDonald diagnostic criteria	ELISA
Weller et al 1991	INF-γ,IL-6,IL-2R	Germany	20/10	NA	NA	NA	NA	BLOOD/CSF	NA	ELISA
Wen et al 2012	IL-6,IL-17, IL-23, TNF-α	China	51/48	29/44	36.49/35.2	6	3.82	BLOOD/CSF	the McDonald criteria	ELISA
WIESEMANN et al 2003	IL-5,IL-13	Germany	63/25	32/24	40/40	NA	6.57	BLOOD	Poser criteria	ELISA
Wing et al 2015	IL-10, IL-4, IL-21, IL-6, IFN-γ, IL-	Brazil	22/22	27/32	27/23.6	0.95	0.75	BLOOD	the McDonald 2010 criteria	ELISA

	21, IL-22, IL-17									
Witkowska et al 2015	TNF-α, sIL-2Rα	Poland	92/30	39/36.7	40.8/39	5.7	3.4	BLOOD	the revised McDonald Diagnostic Criteria	ELISA
Wu et al 2012	IL-21	China	20/16	35/44	35.5/34	3.5	2.3	CSF	2010 McDonald’s criteria	ELISA
Wullschleger et al 2013	IL-6	Germany	76/212	39/NA	37.3/NA	3.53	2.8	CSF	the revised 2005 McDonald Criteria	ELISA
Xu et al 2013	IL-22, IFN-γ, IL-6, IL-21 IL-27.	Australia	15/12	33/25	35.33/35.25	3.87	3.43	BLOOD	the 2005 McDonald criteria	ELISA
Yang et al 2016	IL-1β TNF-α	China	25/20	24/25	33.84/32.3	NA	1.92	BLOOD	2010 revised McDonald criteria	MILLIPLEX® map Human High Sensitivity Cytokine/Chemokine Panels
Yeung et al 2016	IL-6, IL-17A	USA	NA	NA	NA	NA	NA	BLOOD	NA	ELISA
Zhang et al 2015	IL-11	USA	59/78	31/30	41/41	NA	NA	BLOOD/CSF	NA	ELISA
Zhen et al 2017	IL-22	China	30/30	NA	NA	NA	NA	BLOOD	NA	ELISA
Zhong et al 2011	CXCL13	China	18/12	39/42	35.5/32.5	4	2.75	CSF	2010 McDonald's criteria	ELISA

eTable1b: Characteristics of included studies measuring blood and CSF cytokine concentrations (within-group)

Study/Year	Cytokines Measured	Country	Samples (MS)	Gender (%Male) (MS)	Mean Age (MS)	MeanMS duration	Diseasestatus severity/EDSS	Sample Source	Diagnosis	Assay type	Medication

Bahner et al 2002	IL-12P40, IL-12P70	Germany	18	67	47	NA	4.5	BLOOD	modified Poser criteria	ELISA	IFNβ-1b
Ashtari et al 2015	IL-10	Iran	44	20.5	31.5	4.1	1.7	BLOOD	McDonald criteria	ELISA	Vitamin D
Bartosik-Psujek et al 2004	CCL2, CCL5	Poland	30	43	31.1	3.5	3.3	BLOOD	McDonald criteria	ELISA	methylprednisolone (MP)
Bartosik-Psujek et al 2004	IL-8	Poland	25	28	34.2	6.4	3	CSF/BLOOD	McDonald criteria	ELISA	Cladribine
Bartosik-Psujek et al 2005	IL-10,IL-12	Poland	29	31	26	7	2.6	CSF/BLOOD	Poser's criteria	ELISA	Avonex,Betaferon
Buttmann et al 2004	CXCL10, CCL2	Germany	62	25	41.6	9.08	2.1	BLOOD	McDonald criteria	ELISA	GA/IFN-β
Campbell et al 2010	IL-8	UK	7	NA	NA	NA	NA	BLOOD	NA	cytometric bead array (CBA)	IFN-β
Comini-Frota et al 2011	CXCL10,CCL2,CCL4,CCL5 CXCL9	Brazil	17	24	38	5	2	BLOOD	the McDonald criteria	ELISA	IFNβ
Dhib-Jalbut et al 2013	IFN-γ,TNF-α,IL-27,TGF-β,IL-10	USA	30	33	36.7	NA	NA	BLOOD	NA	ELISA	IFNβ-1b
Dressel et al 2006	IL-10	Germany	20	67	46.8	NA	4	BLOOD	NA	ELISA	IFN-β1b
Fan et al 2015	IL-21	China	25	32	40	2	2	BLOOD	the 2010 McDonald's diagnostic	ELISA	methylprednisolone
Festa et al 2009	CXCL13	USA	74	31	36	1	2	BLOOD	NA	ELISA	IFNβ1b;GA
Graber et al 2007	IL-10 IL-12P70	USA	15	NA	NA	2	0-5.5	BLOOD	Poser criteria	ELISA	IFN-β
Grau-López et al 2015	IL-4,IL-6, IL-10,IFNγ	Spain	39	NA	NA	NA	0-5.5	BLOOD	the 2005 McDonald criteria	ELISA	methylprednisolone
Hartung et al 2013	IL-17F	German	239	28	36.5	5.3	23	BLOOD	NA	Immunoassay	interferon beta-1b
Hedegaard et al 2010	BAFF	Denmark	49	35	32.65	2.42	1.69	BLOOD	NA	ELISA	IFN-β1a
Hegen et al 2016	IL–1β,IL-8,CXCL10, CCL2	Austria	157	31.2	36.4	4.8	NA	BLOOD	NA	Luminex assays	IFN-beta

	sTRAIL										
Holm øy et al 2013	PTX3,sTNF-R1,CXCL16,MMP-9,CCL21,IL-1Ra,OPN,OPG TGFβ1,ALCAM	Norway	85	35	38.7	1.9	1.9	BLOOD	the McDonald criteria	enzyme immunoassay (EIA)	ω-3 fatty acids
iseri et al 2013	TNF-α	Turkey	12	NA	30.4	5.3	3	BLOOD	MacDonald criteria	ELISA	methylprednisolone
Jensen et al 2004	TNF IL-12p40	Denmark	10	40	41	7	3.5	BLOOD	NA	ELISA	IFN-β1b
Kalinowska-Lyszczarz et al 2011	CCL17 IL-17	Poland	15	NA	NA	NA	NA	BLOOD	revised McDonald criteria	ELISA	methylprednisolone
Katona et al 2005	IFN-γ,IL-10,IL-12,CRP	UK	100	25	52	NA	NA	BLOOD	NA	ELISA	Cannador,Marinol
Kumpfel et al 2007	TNF-α IL-10,IL-6, sTNF-RII	Germany	16	31.25	35	NA	1.5	BLOOD	the McDonald criteria	ELISA	IFN-β
Kurne et al 2010	BAFF sTRAIL	Turkey	35	40	25.4	6.05	2.4	BLOOD	McDonald criteria	ELISA	IFN-β
Kurtuncu et al 2012	IL-17,IL-23IL-10,IL-4, IFN-γ,IL-9 TGFβ	Turkey	63	32	35.2	3.9	1.9	BLOOD	the McDonald criteria	ELISA	IFN-β
Kvarnstrom et al 2013	IFN-γ,IL-13IL-10 IL-17A	Sweden	25	28	46	13	2	BLOOD	the McDonald criteria	Multiple bead technology	IFN-β
Lomakin et al 2016	TNF-α,IFN-γ,CCL4,CCL2,G-CSF,IL-1β,IL-2,IL-4,IL-5,IL-6,IL-7,IL-8,IL-10,IL-12,IL-13,IL-17,GM-CSF	Russia	20	55	37.6	NA	4	BLOOD	the McDonald diagnostic criteria in 2005	multiplexed fluorescent magnetic bead-based immunoassay	encapsulated MBP peptides
Losy et al 2002	IL-12 TGF-β1	Poland	20	20	32.6	4.4	2.6	BLOOD	Poser criteria	ELISA	IFN-β1a
Losy et al 2002	IL-10,IL-12	Poland	31	35	34	5	2.7	BLOOD	Poser criteria	ELISA	GA

Lund et al 2004	CXCL8	US	20	5	39.85	4.75	1.925	BLOOD	Poser criteria	cytometric bead array (CBA)	Interferon-β1a
Lus et al 2009	TNF-α	Italy	26	NA	33	5	1.15	BLOOD	McDonald criteria	ELISA	Interferon-β1a
Mellergard et al 2012	IL-1β,IL-6 TNF,CXCL8 CXCL10 CXCL11 CCL22	Sweden	25	52	40	9.3	2.5	CSF	the McDonald criteria	ultra-sensitive 10-plex multiple bead kit	Natalizumab
Milosevic et al 2015	IL-17A IL-17F	Serbia	37	27	36.1	7.7	0-3.5	BLOOD	McDonald criteria	ELISA	IFN-β1b
Napolitano et al 2014	CRP	Italy	110	32	25–72	NA	NA	BLOOD	NA	immunoturbidimetric method	Endovascular treatment
Nicolleti et al 2000	IL-1β,IL-2,IL-4,IL-6,IL-10,INF-γ TNF-α	Italy	14	NA	28	6.5	1.9	BLOOD	Poser criteria	ELISA	IFN-β1b
Noroozi et al 2016	IL-1β,IL-18	Iran	30	27	NA	NA	NA	BLOOD	revised MacDonald criteria	ELISA	IFN-β 1a
Novakova et al 2016	CXCL13 CCL2	Sweden	43	37.21	39.65	NA	2.5	CSF	revised MacDonald criteria	ELISA	Fingolimod
Orefice et al 2016	IFN-γ,IL-17,TNF-α	Italy	15	60	30.6	NA	1.7	BLOOD	McDonald's 2010 criteria	ELISA	IFN-β1a+ultramicronized PEA
Piccio et al 2010	CCL2,CCL4,CXCL10,CXCL12,CXCL16, CCL19,CXCL13,IL-16	USA	23	27	43.2	10.8	4	CSF/BLOOD	NA	ELISA	rituximab
Ramirez-Ramirez et al 2013	TNF-α IL-1b,IL-6	Mexico	25	83.4	35.1	7.14	2.1	BLOOD	NA	ELISA	IFNβ-1b,Fish oil
Ramos-Cejudo et al 2011	IL-1α IL-1β IL-2 IL-4 IL-5 IL-6 IL-8 IL-10 IL-12p70 IL-13 IL-17 IFN-γ TNF-α	Spain	22	23	35.53	9.95	3.69	BLOOD	the McDonald criteria	flow cytometry	natalizumab
Rentzos et al 2008	IL-10,IL-12CCL2	Greece	20	35	33.75	8.35	1.67	BLOOD	the criteria of McDonald	ELISA	steroid

Rentzos et al 2010	IL-15	Greece	24	37.5	34	5	3	BLOOD	the criteria of McDonald	ELISA	IVMP
Salama et al 2003	TNF-α,IL-2R,IL-10, IL-12	USA	55	32.73	39.42	8.7	5.94	BLOOD	NA	ELISA	Beta-IFN-1a, prednisone
Sanoobar et al 2014	TNF-α, IL-6 ,TGF-βIL-4 MMP-9	Iran	22	9	33.1	4.4	1.88	BLOOD	the McDonald criteria	ELISA	coenzyme Q10
Seledtsova et al 2016	IFNγ,IL-17,IL-18,IL-4 IL-10	Russia	39	27	35	9.44	0-10	BLOOD	NA	ELISA	T-cell vaccination
Sellebjerg et al 2009	CXCL13	Denmark	20	43	35.72	NA	NA	CSF	NA	ELISA	natalizumab
Sellner et al 2008	IL-1β IL-2 IL-6 IL-12p70 TNF-α IFN-γ IL-4 IL-5 IL-10	Switzerland	24	NA	31.6	3.5	2	BLOOD	the criteria of McDonald	the human LINCOplex High Sensitivity Cytokine kit	IFN-β
Szczucinski et al 2010	CCL5 CXCL10 CXCL11	Poland	17	65	34.8	2.7	2.9	BLOOD/CSF	Mc Donald criteria	ELISA	methylprednisolone (MP)
Stepien et al 2013	TNFα,IFNγ,IL-10,IL-6	Denmark	38	34	33	2	1.7	BLOOD	Mc Donald criteria	ELISA	IFNβ
Toghianifar et al 2015	IL-17	Iran	44	20.5	31.5	4.1	1.7	BLOOD	Mc Donald criteria	ELISA	Vitamin D
Tumani et al 2011	IFN-γ,IL-2, IL-4,IL-10	Germany	19	32	34	1.3	2	BLOOD	the revised McDonald criteria	electrochemiluminescence detection multiplex technology	GA(glatiramer acetate)
Valenzuela et al 2016	IL-17 IFN-γ TNFα IL-18 IL-4 TGF-β IL-10	USA	32	21.9/	41.2/	9.8	2.06	BLOOD	2010 revised McDonald criteria	ELISA	GA(glatiramer acetate)

eTable 2. Newcastle-Ottawa quality assessment scale for case-control (between group) and cohort (within-group) studies.

Studies	Selection	Comparability	Exposure	Total
Abraham et al., 2017 ()	*	*	**	4
Adachi et al., 1990	***	*	*	5
Akcali et al., 2017 ()	***	*	**	6

Alexander et al., 2010	***	**	**	7
Alsahebfosoul *et al.,* 2017 ()	***	**	*	6
Altıokka-Uzun et al., 2015	***	**	**	7
Alvarez et al., 2013 ()	***	**	*	6
Arababadi et al., 2010	****	**	*	7
Ashtari et al., 2015	**	*	**	5
Babaloo et al., 2015	***	*	*	5
Bahner *et al.,* 2002 ()	**	*	**	5
Bălaşa et al., 2015	***	*	**	6
Bansi et al., 1991	***	*	*	5
Bartosik-Psujek *et al.,* 2004 ()	**	*	*	4
Bartosik-Psujek *et al.,* 2004 ()	***	**	*	6
Bartosik-Psujek *et al.,* 2005 ()	***	*	*	5
Bartosik-Psujek *et al.,* 2005 ()	***	*	*	5
Bielekova et al., 2012	***	*	**	6
Bonin et al., 2017 ()	**	*	**	5
Burman et al., 2014 ()	***	*	*	5
Buttmann *et al.,* 2004 ()	***	*	*	5
Cala et al., 2016	***	*	**	6
Campbell et al., 2010	***	**	*	6
Castellano *et al.,* 2008 ()	**	*	*	4
Chalon et al., 1992	***	*	*	5
Chen et al., 2012	***	**	**	7
Chen et al., 2012 ()	***	**	*	6
Christensen et al., 2012 ()	***	*	**	6
Christophi et al., 2011 （）	***	*	**	6
Christophi et al., 2012	***	*	*	5
Comini-Frota et al., 2011 ()	***	*	**	6
Damasceno et al., 2016	***	*	*	5
de Flon et al., 2018 ()	***	*	**	6
de J. Guerrero-García et al., 2017 ()	****	**	**	8
Dhib-Jalbut et al., 2013	**	0	**	4
Dressel *et al.,* 2006 ()	**	---	**	4
Drulovic et al., 1998	***	*	*	5
DruloviC" et al., 1997	***	*	*	5
Duddy et al., 1999	***	**	**	7
Edwards et al., 2013	***	*	**	6
Emamgholipour et al., 2013 ()	****	**	*	7
Esendagli et al., 2013	***	**	**	7
Fan et al., 2015	****	**	**	8
Farhadi et al., 2013 ()	***	*	*	5
Farrokhi et al., 2015 ()	**	**	*	5
Farrokhi et al., 2015 ()	**	**	*	5

Farrokhi *et al.,* 2017 ()	***	**	*	6
Fassbender et al., 1998	***	**	**	7
Festa *et al.,* 2009 ()	**	*	**	5
Fjeldstad et al., 2011	***	**	**	7
Franciotta *et al.,* 1989 ()	**	*	*	4
Franciotta *et al.,* 2006 ()	**	*	*	4
Galimberti *et al.,* 2008	***	**	*	6
Gallo et al., 1988	**	*	*	4
Gallo et al., 1989	**	*	*	4
Giunti *et al.,* 2003 ()	***	*	*	5
Glasnović et al., 2014	***	*	**	6
Graber *et al.,* 2007 ()	**	---	**	4
Grau-López et al., 2015	**	*	**	5
Guerrero-García et al., 2016	****	**	*	7
Haas et al., 2017	***	**	**	7
Hagman et al., 2011	***	*	**	6
Håkansson *et al.,* 2017 ()	***	**	**	7
Hartung et al., 2013 ()	***	*	*	5
Hashemi *et al.,* 2006 ()	***	*	*	5
Hedegaard *et al.,* 2010 ()	**	---	**	4
Hegen *et al.,* 2016 ()	**	---	***	5
Heesen et al., 1999	***	*	*	5
Hietaharju et al., 2010	***	*	**	6
Hohnoki et al., 1998	**	*	*	4
Hollifield *et al.,* 2003 ()	***	*	*	5
Holmøy et al., 2013	**	*	**	5
Hornig et al., 2016	***	**	**	7
Huber et al., 2014 ()	***	**	***	8
İşeri et al., 2013 ()	**	---	**	4
Jafarzadeh et al., 2013 ()	***	*	*	5
Jafarzadeh et al., 2014	****	*	**	7
Jafarzadeh et al., 2014	****	*	**	7
Jafarzadeh *et al.,* 2016 ()	***	**	*	6
Jensen *et al.,* 2005 ()	**	*	**	5
Ji *et al.,* 2016 ()	***	*	*	5
Kalinowska-Łyszczarz et al. 2011（）	***	**	*	6
Kallaur et al., 2013 ()	***	**	*	6
Kallaur et al., 2016	****	*	**	7
Kallaur *et al.,* 2016 ()	***	**	*	6
Kannel et al., 2015 ()	***	*	**	6
Katona *et al.,* 2005 ()	***	*	**	6
Khademi *et al.,* 2010 ()	****	*	*	6
Khaiboullina et al., 2015	***	*	**	6

Kittur et al., 1990	***	*	*	5
Kostic et al., 2014	***	**	**	7
Kowarik et al., 2012	***	*	**	6
Kreft et al., 2012 ()	***	*	*	5
Kümpfel *et al.,* 2007 ()	**	---	***	5
Kurne *et al.,* 2010 ()	**	**	**	6
Kürtüncü et al., 2012	***	*	*	5
Kvarnström et al., 2013 ()	***	*	*	5
Lebrun et al., 2016	**	*	*	4
Lomakin et al., 2016				0
Losy *et al.,* 2002 ()	***	*	**	6
Losy *et al.,* 2002 ()	***	**	**	7
Lund *et al.,* 2004 ()	***	*	*	5
Lundström et al., 2014 ()	***	*	**	6
Lus *et al.,* 2009 ()	**	*	*	4
Mahad *et al.,* 2001 ()	***	**	**	7
Maimone et al., 1991	**	*	*	4
Malekzadeh *et al.,* 2017 ()	***	*	**	6
Malmeström *et al.,* 2006 ()	***	*	*	5
Martínezcáceres *et al.,* 2002 ()	***	**	**	7
Martins et al., 2011	***	**	**	7
Matejčíková et al., 2014 ()	**	*	**	5
Matejčíková et al., 2017 ()	**	**	**	6
Matsushita et al., 2013	***	**	**	7
Mellergard et al., 2010	***	*	**	6
Mellergård et al., 2012 ()	***	*	*	5
Michalopoulou *et al.,* 2004 ()	***	*	*	5
Michałowskawender *et al.,* 2008 ()	***	*	*	5
Mikulkova et al., 2011	****	**	**	8
Miljkovic *et al.,* 2002 ()	***	*	**	6
Milosevic *et al.,* 2015 ()	**	---	***	5
Mirandola *et al.,* 2009 ()	****	*	*	6
Mori et al., 2016	***	*	*	5
Morsaljahan et al., 2017 ()	***	**	**	7
Mouzaki et al., 2015	***	**	**	7
Muls *et al.,* 2017 ()	***	*	*	5
Musabak et al., 2011	***	**	**	7
Naderi *et al.,* 2016 ()	****	**	**	8
Napolitano et al., 2014	***	*	**	6
Narikawa *et al.,* 2005 ()	***	*	*	5
Nicoletti et al., 1996	**	**	*	5
Nicoletti et al., 1998	***	*	*	5

Nicolleti et al., 2000	***	**	*	6
Niedziela *et al.,* 2016 ()	***	**	*	6
Nischwitz et al., 2014	***	**	*	6
Noroozi *et al.,* 2016 ()	**	---	***	5
Novakova *et al.,* 2016 ()	***	**	**	7
Novakova et al., 2017	***	*	**	6
Obradović et al., 2012	***	**	**	7
Oliveira *et al.,* 2016 ()	***	*	*	5
Oliveira et al., 2017 ()	****	**	**	8
Orefice *et al.,* 2016	***	**	***	7
Orhan et al., 2016	***	**	**	7
Padberg et al., 1999	***	*	*	5
Paroni *et al.,* 2017 ()	***	**	**	7
Pashenkov *et al.,* 2003 ()	***	*	*	5
Perriard et al., 2015	***	*	**	6
Piazza *et al.,* 2010 ()	***	**	*	6
Piccio *et al.,* 2010 ()	***	---	**	5
Polachini et al., 2014（）	***	*	*	5
Puthenparampil *et al.,* 2016 ()	***	**	**	7
Puthenparampil *et al.,* 2017 ()	***	**	**	7
Ragheb et al., 2011	***	*	*	5
Ramirez-Ramirez et al., 2013	**	**	*	5
Ramos-Cejudo et al., 2011	****	*	**	7
Reale et al., 2015	**	*	*	4
Rentzos et al., 1996	***	*	*	5
Rentzos *et al.,* 2006 ()	***	*	*	5
Rentzos *et al.,* 2008 ()	*	---	*	2
Rentzos et al., 2010	***	**	*	6
Rollnik et al., 1997	***	*	*	5
Rossi et al., 2011	***	**	*	6
Rossi et al., 2013 ()	**	**	**	6
Rossi et al., 2015	***	**	**	7
Rudick et al., 1990	**	*	*	4
Rudick et al., 1990	**	*	*	4
Ruocco et al., 2015	***	**	*	6
Sadowska-Bartosz et al., 2013 ()	***	*	**	6
Salama *et al.,* 2003 ()	***	**	*	6
Salehi et al., 2016	***	**	*	6
Salmaggi et al., 1995	***	*	**	6
Sanoobar et al., 2015 ()	**	*	**	5
Saruhan-Direskeneli *et al.,* 2003 ()	***	*	*	5
Sayad et al., 2014 ()	**	**	*	5
Sayad et al., 2014 ()	**	**	*	5

Scalabrino et al., 2010	***	*	*	5
Seledtsova et al., 2016				0
Sellebjerg *et al.,* 2009 ()	***	**	*	6
Sellner *et al.,* 2008 ()	*	---	**	3
Sexton et al., 2014	***	*	*	5
Shajarian et al., 2014 ()	***	**	*	6
Sharief et al., 1991	***	**	*	6
Shu et al., 2017 ()	**	*	**	5
Sørensen et al., 1999	***	*	**	6
Sørensen *et al.,* 2004 ()	***	*	*	5
Stępień et al., 2013	**			2
Stojkovic´ et al., 2014 ()	****	**	*	7
Su et al., 2014	***	**	*	6
Sumita et al., 2012	*	*	*	3
Szczuciński et al., 2011	***	**	*	6
Tanaka *et al.,* 2008 ()	***	**	*	6
Tang et al., 2015	***	*	*	5
Tao et al., 2014	***	*	*	5
Tawfik et al., 2016	**	**	*	5
Tejera-Alhambra et al., 2015	****	**	**	8
Tiumentseva et al., 2016	***	**	*	6
Toghianifar *et al.,* 2015 ()	**	*	**	5
Tomioka et al., 1992	**	*	*	4
Tong et al., 2018 ()	**	**	**	6
Trenova et al., 2014	***	**	*	6
Trenova *et al.,* 2017 ()	***	**	*	6
Trotter et al., 1989	***	**	**	7
Trotter et al., 1990	**	**	*	5
Trotter et al., 1991	***	**	**	7
Tsukada et al., 1991	***	*	**	6
Tsukada et al., 1991	***	*	**	6
Tumani et al., 2011 ()	**	**	*	5
Uzawa et al., 2010	***	*	**	6
Valenzuela *et al.,* 2016 ()	**	---	***	5
Vrethem et al., 2012	*	**	**	5
Wang et al., 2012 ()	***	**	*	6
Wang et al., 2013 ()	***	*	*	5
Wang et al., 2017 ()	**	**	**	6
Wang et al., 2017 ()	**	*	**	5
Weller et al., 1991	***	**	**	7
Wen et al., 2012	***	**	*	6
Wiesemann *et al.,* 2003 ()	***	**	**	7
Wing *et al.,* 2015 ()	***	*	**	6

Witkowska *et al.,* 2015 ()	**	**	*	5
Wu et al., 2012 ()	***	**	*	6
Wullschleger et al., 2013	***	*	*	5
Xu et al., 2013 ()	***	**	*	6
Yang et al., 2016	***	*	*	5
Yeung et al., 2016	**	*	**	5
Zhang et al., 2015	***	*	**	6
Zhen *et al.,* 2017 ()	**	*	**	5
Zhong et al., 2011	***	**	*	6

eReference: 226 included articles in the meta-analysis

1. Abraham M, Karni A, Mausner-Fainberg K, Weiss ID, Peled A. Natural and induced immunization against CCL20 ameliorate experimental autoimmune encephalitis and may confer protection against multiple sclerosis. *Clinical immunology.* Oct 2017;183:316-324.
2. Adachi K, Kumamoto T, Araki S. Elevated soluble interleukin-2 receptor levels in patients with active multiple sclerosis. *Annals of neurology.* Nov 1990;28(5):687-691.
3. Akcali A, Zengin F, Aksoy SN, Zengin O. Fatigue in Multiple Sclerosis: Is it related to cytokines and hypothalamic-pituitary-adrenal axis? *Multiple sclerosis and related disorders.* Jul 2017;15:37-41.
4. Alexander JS, Harris MK, Wells SR, et al. Alterations in serum MMP-8, MMP-9, IL-12p40 and IL-23 in multiple sclerosis patients treated with interferon-beta1b. *Multiple sclerosis.* Jul 2010;16(7):801-809.
5. Alsahebfosoul F, Rahimmanesh I, Shajarian M, et al. Interleukin-33 plasma levels in patients with relapsing-remitting multiple sclerosis. *Biomolecular concepts.* Mar 1 2017;8(1):55-60.
6. Altiokka-Uzun G, Tuzun E, Ekizoglu E, et al. Oligoclonal bands and increased cytokine levels in idiopathic intracranial hypertension. *Cephalalgia : an international journal of headache.* Nov 2015;35(13):1153-1161.
7. Alvarez E, Piccio L, Mikesell RJ, et al. CXCL13 is a biomarker of inflammation in multiple sclerosis, neuromyelitis optica, and other neurological conditions. *Multiple sclerosis.* Aug 2013;19(9):1204-1208.
8. Arababadi MK, Mosavi R, Khorramdelazad H, et al. Cytokine patterns after therapy with Avonex(R), Rebif(R), Betaferon(R) and CinnoVex in relapsing-remitting multiple sclerosis in Iranian patients. *Biomarkers in medicine.* Oct 2010;4(5):755-759.
9. Ashtari F, Toghianifar N, Zarkesh-Esfahani SH, Mansourian M. Short-term effect of high-dose vitamin D on the level of interleukin 10 in patients with multiple sclerosis: a randomized, double-blind, placebo-controlled clinical trial. *Neuroimmunomodulation.* 2015;22(6):400-404.
10. Babaloo Z, Aliparasti MR, Babaiea F, Almasi S, Baradaran B, Farhoudi M. The role of Th17 cells in patients with relapsing-remitting multiple sclerosis: interleukin-17A and interleukin-17F serum levels. *Immunology letters.* Apr 2015;164(2):76-80.
11. Bahner D, Klucke C, Kitze B, et al. Interferon-beta-1b increases serum interleukin-12 p40 levels in primary progressive multiple sclerosis patients. *Neuroscience letters.* Jun 28 2002;326(2):125-128.
12. Balasa R, Maier S, Voidazan S, et al. Assessment of Interleukin-17A, Interleukin-10 and Transforming Growth Factor-Beta1 Serum Titers in Relapsing Remitting Multiple Sclerosis Patients Treated with Avonex, Possible Biomarkers for Treatment Response. *CNS & neurological disorders drug targets.* 2017;16(1):93-101.
13. Bansil S, Troiano R, Cook SD, Rohowsky-Kochan C. Serum soluble interleukin-2 receptor levels in chronic progressive, stable and steroid-treated multiple sclerosis. *Acta neurologica Scandinavica.* Oct 1991;84(4):282-285.

14. Bartosik-Psujek H, Belniak E, Mitosek-Szewczyk K, Dobosz B, Stelmasiak Z. Interleukin-8 and RANTES levels in patients with relapsing-remitting multiple sclerosis (RR-MS) treated with cladribine. *Acta neurologica Scandinavica.* Jun 2004;109(6):390-392.

15. Bartosik-Psujek H, Stelmasiak Z. Steroid therapy altered serum levels of CCL2 and CCL5 chemokines in multiple sclerosis patients during relapse. *European neurology.* 2004;52(4):237-241.

16. Bartosik-Psujek H, Stelmasiak Z. Correlations between IL-4, IL-12 levels and CCL2, CCL5 levels in serum and cerebrospinal fluid of multiple sclerosis patients. *Journal of neural transmission.* Jun 2005;112(6):797-803.

17. Bartosik-Psujek H, Stelmasiak Z. The levels of chemokines CXCL8, CCL2 and CCL5 in multiple sclerosis patients are linked to the activity of the disease. *European journal of neurology.* Jan 2005;12(1):49-54.

18. Bielekova B, Komori M, Xu Q, Reich DS, Wu T. Cerebrospinal fluid IL-12p40, CXCL13 and IL-8 as a combinatorial biomarker of active intrathecal inflammation. *PloS one.* 2012;7(11):e48370.

19. Bonin S, Zanotta N, Sartori A, et al. Cerebrospinal Fluid Cytokine Expression Profile in Multiple Sclerosis and Chronic Inflammatory Demyelinating Polyneuropathy. *Immunological investigations.* Feb 2018;47(2):135-145.

20. Braun Hashemi CA, Zang YC, Arbona JA, et al. Serum immunologic markers in multiple sclerosis patients on continuous combined therapy with beta-interferon 1a, prednisone and azathioprine. *Multiple sclerosis.* Oct 2006;12(5):652-658.

21. Burman J, Svensson E, Fransson M, et al. The cerebrospinal fluid cytokine signature of multiple sclerosis: a homogenous response that does not conform to the Th1/Th2/Th17 convention. *Journal of neuroimmunology.* Dec 15 2014;277(1-2):153-159.

22. Buttmann M, Merzyn C, Rieckmann P. Interferon-beta induces transient systemic IP-10/CXCL10 chemokine release in patients with multiple sclerosis. *Journal of neuroimmunology.* Nov 2004;156(1-2):195-203.

23. Cala CM, Moseley CE, Steele C, et al. T cell cytokine signatures: Biomarkers in pediatric multiple sclerosis. *Journal of neuroimmunology.* Aug 15 2016;297:1-8.

24. Campbell SJ, Meier U, Mardiguian S, et al. Sickness behaviour is induced by a peripheral CXC-chemokine also expressed in multiple sclerosis and EAE. *Brain, behavior, and immunity.* Jul 2010;24(5):738-746.

25. Capra R, Mattioli F, Marciano N, et al. Significantly higher levels of soluble interleukin 2 in patients with relapsing-remitting multiple sclerosis compared with healthy subjects. *Archives of neurology.* Mar 1990;47(3):254.

26. Castellano V, Patel DI, White LJ. Cytokine responses to acute and chronic exercise in multiple sclerosis. *Journal of applied physiology.* Jun 2008;104(6):1697-1702.

27. Chalon MP, Sindic CJ, Laterre EC. Serum and CSF levels of soluble interleukin-2 receptors in MS and other neurological diseases: a reappraisal. *Acta neurologica Scandinavica.* Feb 1993;87(2):77-82.

28. Chen YC, Chen SD, Miao L, et al. Serum levels of interleukin (IL)-18, IL-23 and IL-17 in Chinese patients with multiple sclerosis. *Journal of neuroimmunology.* Feb 29 2012;243(1-2):56-60.

29. Chen YC, Yang X, Miao L, et al. Serum level of interleukin-6 in Chinese patients with multiple sclerosis. *Journal of neuroimmunology.* Aug 15 2012;249(1-2):109-111.

30. Christophi GP, Christophi JA, Gruber RC, et al. Quantitative differences in the immunomodulatory effects of Rebif and Avonex in IFN-beta 1a treated multiple sclerosis patients. *Journal of the neurological sciences.* Aug 15 2011;307(1-2):41-45.

31. Christophi GP, Gruber RC, Panos M, Christophi RL, Jubelt B, Massa PT. Interleukin-33 upregulation in peripheral leukocytes and CNS of multiple sclerosis patients. *Clinical immunology.* Mar 2012;142(3):308-319.

32. Comini-Frota ER, Teixeira AL, Angelo JP, et al. Evaluation of serum levels of chemokines during interferon-beta treatment in multiple sclerosis patients: a 1-year, observational cohort study. *CNS drugs.* Nov 1 2011;25(11):971-981.

33. Damasceno A, Moraes AS, Farias A, Damasceno BP, dos Santos LM, Cendes F. A spring to summer shift of pro-

inflammatory cytokine production in multiple sclerosis patients. *Journal of the neurological sciences.* Jan 15 2016;360:37-40.

34. de Flon P, Soderstrom L, Laurell K, et al. Immunological profile in cerebrospinal fluid of patients with multiple sclerosis after treatment switch to rituximab and compared with healthy controls. *PloS one.* 2018;13(2):e0192516.

35. de JG-GJ, Rojas-Mayorquin AE, Valle Y, et al. Decreased serum levels of sCD40L and IL-31 correlate in treated patients with Relapsing-Remitting Multiple Sclerosis. *Immunobiology.* Jan 2018;223(1):135-141.

36. Dhib-Jalbut S, Sumandeep S, Valenzuela R, Ito K, Patel P, Rametta M. Immune response during interferon beta-1b treatment in patients with multiple sclerosis who experienced relapses and those who were relapse-free in the START study. *Journal of neuroimmunology.* Jan 15 2013;254(1-2):131-140.

37. Dressel A, Kolb AK, Elitok E, et al. Interferon-beta1b treatment modulates cytokines in patients with primary progressive multiple sclerosis. *Acta neurologica Scandinavica.* Dec 2006;114(6):368-373.

38. Drulovic J, Mostarica-Stojkovic M, Levic Z, et al. Serum interleukin-12 levels in patients with multiple sclerosis. *Neuroscience letters.* Jul 24 1998;251(2):129-132.

39. Drulovic J, Mostarica-Stojkovic M, Levic Z, Stojsavljevic N, Pravica V, Mesaros S. Interleukin-12 and tumor necrosis factor-alpha levels in cerebrospinal fluid of multiple sclerosis patients. *Journal of the neurological sciences.* Apr 15 1997;147(2):145-150.

40. Duddy ME, Armstrong MA, Crockard AD, Hawkins SA. Changes in plasma cytokines induced by interferon-beta1a treatment in patients with multiple sclerosis. *Journal of neuroimmunology.* Nov 1 1999;101(1):98-109.

41. Edwards KR, Goyal J, Plavina T, et al. Feasibility of the use of combinatorial chemokine arrays to study blood and CSF in multiple sclerosis. *PloS one.* 2013;8(11):e81007.

42. Emamgholipour S, Eshaghi SM, Hossein-nezhad A, Mirzaei K, Maghbooli Z, Sahraian MA. Adipocytokine profile, cytokine levels and foxp3 expression in multiple sclerosis: a possible link to susceptibility and clinical course of disease. *PloS one.* 2013;8(10):e76555.

43. Esendagli G, Kurne AT, Sayat G, Kilic AK, Guc D, Karabudak R. Evaluation of Th17-related cytokines and receptors in multiple sclerosis patients under interferon beta-1 therapy. *Journal of neuroimmunology.* Feb 15 2013;255(1-2):81-84.

44. Fan X, Jin T, Zhao S, et al. Circulating CCR7+ICOS+ Memory T Follicular Helper Cells in Patients with Multiple Sclerosis. *PloS one.* 2015;10(7):e0134523.

45. Farhadi N, Oryan S, Nabiuni M. Serum levels of melatonin and cytokines in multiple sclerosis. *Biomedical journal.* Mar-Apr 2014;37(2):90-92.

46. Farrokhi M, Etemadifar M, Jafary Alavi MS, et al. TNF-alpha Production by Peripheral Blood Monocytes in Multiple Sclerosis Patients and Healthy Controls. *Immunological investigations.* 2015;44(6):590-601.

47. Farrokhi M, Masoudifar A, Derakhshan A, et al. The Association of Interleukin-16 Gene Polymorphisms with IL-16 Serum Levels and Risk of Multiple Sclerosis. *Immunological investigations.* Feb 2 2017:1-9.

48. Farrokhi M, Rezaei A, Amani-Beni A, Etemadifar M, Kouchaki E, Zahedi A. Increased serum level of IL-37 in patients with multiple sclerosis and neuromyelitis optica. *Acta neurologica Belgica.* Dec 2015;115(4):609-614.

49. Fassbender K, Ragoschke A, Rossol S, et al. Increased release of interleukin-12p40 in MS: association with intracerebral inflammation. *Neurology.* Sep 1998;51(3):753-758.

50. Festa ED, Hankiewicz K, Kim S, et al. Serum levels of CXCL13 are elevated in active multiple sclerosis. *Multiple sclerosis.* Nov 2009;15(11):1271-1279.

51. Fjeldstad AS, McDaniel J, Witman MA, et al. Vascular function and multiple sclerosis. *Journal of neurology.* Nov 2011;258(11):2036-2042.

52. Franciotta D, Bergamaschi R, Martino G, Zardini E, Desina G, Cosi V. Tumor necrosis factor-alpha and its soluble receptors in plasma and cerebrospinal fluid of multiple sclerosis patients treated with methylprednisolone.

European cytokine network. Sep 1999;10(3):431-436.

53. Franciotta D, Zardini E, Ravaglia S, et al. Cytokines and chemokines in cerebrospinal fluid and serum of adult patients with acute disseminated encephalomyelitis. *Journal of the neurological sciences.* Sep 25 2006;247(2):202-207.

54. Franciotta DM, Grimaldi LM, Martino GV, et al. Tumor necrosis factor in serum and cerebrospinal fluid of patients with multiple sclerosis. *Annals of neurology.* Dec 1989;26(6):787-789.

55. Galimberti D, Fenoglio C, Comi C, et al. MDC/CCL22 intrathecal levels in patients with multiple sclerosis. *Multiple sclerosis.* May 2008;14(4):547-549.

56. Gallo P, Piccinno M, Pagni S, Tavolato B. Interleukin-2 levels in serum and cerebrospinal fluid of multiple sclerosis patients. *Annals of neurology.* Dec 1988;24(6):795-797.

57. Gallo P, Piccinno MG, Pagni S, et al. Immune activation in multiple sclerosis: study of IL-2, sIL-2R, and gamma-IFN levels in serum and cerebrospinal fluid. *Journal of the neurological sciences.* Aug 1989;92(1):9-15.

58. Giunti D, Borsellino G, Benelli R, et al. Phenotypic and functional analysis of T cells homing into the CSF of subjects with inflammatory diseases of the CNS. *Journal of leukocyte biology.* May 2003;73(5):584-590.

59. Glasnovic A, Cvija H, Stojic M, et al. Decreased level of sRAGE in the cerebrospinal fluid of multiple sclerosis patients at clinical onset. *Neuroimmunomodulation.* 2014;21(5):226-233.

60. Graber JJ, Ford D, Zhan M, Francis G, Panitch H, Dhib-Jalbut S. Cytokine changes during interferon-beta therapy in multiple sclerosis: correlations with interferon dose and MRI response. *Journal of neuroimmunology.* Apr 2007;185(1-2):168-174.

61. Grau-Lopez L, Teniente-Serra A, Tintore M, et al. Similar biological effect of high-dose oral versus intravenous methylprednisolone in multiple sclerosis relapses. *Multiple sclerosis.* Apr 2015;21(5):646-650.

62. Guerrero-Garcia Jde J, Castaneda-Moreno VA, Torres-Carrillo N, et al. Interleukin-17A Levels Vary in Relapsing-Remitting Multiple Sclerosis Patients in Association with Their Age, Treatment and the Time of Evolution of the Disease. *Neuroimmunomodulation.* 2016;23(1):8-17.

63. Haas J, Schneider K, Schwarz A, et al. Th17 cells: A prognostic marker for MS rebound after natalizumab cessation? *Multiple sclerosis.* Jan 2017;23(1):114-118.

64. Hagman S, Raunio M, Rossi M, Dastidar P, Elovaara I. Disease-associated inflammatory biomarker profiles in blood in different subtypes of multiple sclerosis: prospective clinical and MRI follow-up study. *Journal of neuroimmunology.* May 2011;234(1-2):141-147.

65. Hakansson I, Tisell A, Cassel P, et al. Neurofilament light chain in cerebrospinal fluid and prediction of disease activity in clinically isolated syndrome and relapsing-remitting multiple sclerosis. *European journal of neurology.* May 2017;24(5):703-712.

66. Hartung HP, Steinman L, Goodin DS, et al. Interleukin 17F level and interferon beta response in patients with multiple sclerosis. *JAMA neurology.* Aug 2013;70(8):1017-1021.

67. Hedegaard CJ, Sellebjerg F, Krakauer M, Hesse D, Bendtzen K, Nielsen CH. Interferon-beta increases systemic BAFF levels in multiple sclerosis without increasing autoantibody production. *Multiple sclerosis.* May 2011;17(5):567-577.

68. Heesen C, Sieverding F, Buhmann C, Gbadamosi J. IL-1ra serum levels in disease stages of MS--a marker for progression? *Acta neurologica Scandinavica.* Feb 2000;101(2):95-97.

69. Hegen H, Adrianto I, Lessard CJ, et al. Cytokine profiles show heterogeneity of interferon-beta response in multiple sclerosis patients. *Neurology(R) neuroimmunology & neuroinflammation.* Apr 2016;3(2):e202.

70. Hietaharju A, Kuusisto H, Nieminen R, Vuolteenaho K, Elovaara I, Moilanen E. Elevated cerebrospinal fluid adiponectin and adipsin levels in patients with multiple sclerosis: a Finnish co-twin study. *European journal of neurology.* Feb 2010;17(2):332-334.

71. Hohnoki K, Inoue A, Koh CS. Elevated serum levels of IFN-gamma, IL-4 and TNF-alpha/unelevated serum levels of IL-10 in patients with demyelinating diseases during the acute stage. *Journal of neuroimmunology.* Jul 1 1998;87(1-

2):27-32.

72. Hollifield RD, Harbige LS, Pham-Dinh D, Sharief MK. Evidence for cytokine dysregulation in multiple sclerosis: peripheral blood mononuclear cell production of pro-inflammatory and anti-inflammatory cytokines during relapse and remission. *Autoimmunity.* May 2003;36(3):133-141.

73. Holmoy T, Loken-Amsrud KI, Bakke SJ, et al. Inflammation markers in multiple sclerosis: CXCL16 reflects and may also predict disease activity. *PloS one.* 2013;8(9):e75021.

74. Hornig M, Gottschalk G, Peterson DL, et al. Cytokine network analysis of cerebrospinal fluid in myalgic encephalomyelitis/chronic fatigue syndrome. *Molecular psychiatry.* Feb 2016;21(2):261-269.

75. Huber AK, Wang L, Han P, et al. Dysregulation of the IL-23/IL-17 axis and myeloid factors in secondary progressive MS. *Neurology.* Oct 21 2014;83(17):1500-1507.

76. Iseri P, Basyigit I, Ozerdem C, Basyigit H, Efendi H, Yildiz F. The effect of pulse methylprednisolone plus theophylline treatment on clinical, pulmonary and inflammatory markers in relapses of multiple sclerosis. *Balkan medical journal.* Mar 2013;30(1):33-36.

77. Jafarzadeh A, Bagherzadeh S, Ebrahimi HA, et al. Higher circulating levels of chemokine CCL20 in patients with multiple sclerosis: evaluation of the influences of chemokine gene polymorphism, gender, treatment and disease pattern. *Journal of molecular neuroscience : MN.* Jul 2014;53(3):500-505.

78. Jafarzadeh A, Ebrahimi HA, Bagherzadeh S, et al. Lower serum levels of Th2-related chemokine CCL22 in women patients with multiple sclerosis: a comparison between patients and healthy women. *Inflammation.* Apr 2014;37(2):604-610.

79. Jafarzadeh A, Jamali M, Mahdavi R, et al. Circulating levels of interleukin-35 in patients with multiple sclerosis: evaluation of the influences of FOXP3 gene polymorphism and treatment program. *Journal of molecular neuroscience : MN.* Apr 2015;55(4):891-897.

80. Jafarzadeh A, Mahdavi R, Jamali M, Hajghani H, Nemati M, Ebrahimi HA. Increased Concentrations of Interleukin-33 in the Serum and Cerebrospinal Fluid of Patients with Multiple Sclerosis. *Oman medical journal.* Jan 2016;31(1):40-45.

81. Jensen J, Krakauer M, Sellebjerg F. Cytokines and adhesion molecules in multiple sclerosis patients treated with interferon-beta1b. *Cytokine.* Jan 7 2005;29(1):24-30.

82. Ji AL, Liu ZH, Chen WW, Huang WJ. The clinical significance of level changes of hs-CRP, IL-10 and TNF for patients with MS during active and relieving period. *European review for medical and pharmacological sciences.* Oct 2016;20(20):4274-4276.

83. Kalinowska-Lyszczarz A, Szczucinski A, Pawlak MA, Losy J. Clinical study on CXCL13, CCL17, CCL20 and IL-17 as immune cell migration navigators in relapsing-remitting multiple sclerosis patients. *Journal of the neurological sciences.* Jan 15 2011;300(1-2):81-85.

84. Kallaur AP, Oliveira SR, Colado Simao AN, et al. Cytokine profile in relapsingremitting multiple sclerosis patients and the association between progression and activity of the disease. *Molecular medicine reports.* Mar 2013;7(3):1010-1020.

85. Kallaur AP, Oliveira SR, Simao ANC, et al. Cytokine Profile in Patients with Progressive Multiple Sclerosis and Its Association with Disease Progression and Disability. *Molecular neurobiology.* May 2017;54(4):2950-2960.

86. Kallaur AP, Reiche EM, Oliveira SR, et al. Genetic, Immune-Inflammatory, and Oxidative Stress Biomarkers as Predictors for Disability and Disease Progression in Multiple Sclerosis. *Molecular neurobiology.* Jan 2017;54(1):31-44.

87. Kannel K, Alnek K, Vahter L, Gross-Paju K, Uibo R, Kisand KV. Changes in Blood B Cell-Activating Factor (BAFF) Levels in Multiple Sclerosis: A Sign of Treatment Outcome. *PloS one.* 2015;10(11):e0143393.

88. Katona S, Kaminski E, Sanders H, Zajicek J. Cannabinoid influence on cytokine profile in multiple sclerosis. *Clinical*

and experimental immunology. Jun 2005;140(3):580-585.

89. Khademi M, Kockum I, Andersson ML, et al. Cerebrospinal fluid CXCL13 in multiple sclerosis: a suggestive prognostic marker for the disease course. *Multiple sclerosis.* Mar 2011;17(3):335-343.

90. Khaiboullina SF, Gumerova AR, Khafizova IF, et al. CCL27: Novel Cytokine with Potential Role in Pathogenesis of Multiple Sclerosis. *BioMed research international.* 2015;2015:189638.

91. Kittur SD, Kittur DS, Soncrant TT, et al. Soluble interleukin-2 receptors in cerebrospinal fluid from individuals with various neurological disorders. *Annals of neurology.* Aug 1990;28(2):168-173.

92. Kostic M, Dzopalic T, Zivanovic S, et al. IL-17 and glutamate excitotoxicity in the pathogenesis of multiple sclerosis. *Scandinavian journal of immunology.* Mar 2014;79(3):181-186.

93. Kowarik MC, Cepok S, Sellner J, et al. CXCL13 is the major determinant for B cell recruitment to the CSF during neuroinflammation. *Journal of neuroinflammation.* May 16 2012;9:93.

94. Kreft KL, Verbraak E, Wierenga-Wolf AF, et al. Decreased systemic IL-7 and soluble IL-7Ralpha in multiple sclerosis patients. *Genes and immunity.* Oct 2012;13(7):587-592.

95. Kumpfel T, Schwan M, Pollmacher T, et al. Time of interferon-beta 1a injection and duration of treatment affect clinical side effects and acute changes of plasma hormone and cytokine levels in multiple sclerosis patients. *Multiple sclerosis.* Nov 2007;13(9):1138-1145.

96. Kurne A, Guc D, Canpinar H, et al. Analysis of BAFF and TRAIL expression levels in multiple sclerosis patients: evaluation of expression under immunomodulatory therapy. *Acta neurologica Scandinavica.* Jan 2011;123(1):8-12.

97. Kurtuncu M, Tuzun E, Turkoglu R, et al. Effect of short-term interferon-beta treatment on cytokines in multiple sclerosis: significant modulation of IL-17 and IL-23. *Cytokine.* Aug 2012;59(2):400-402.

98. Kvarnstrom M, Ydrefors J, Ekerfelt C, Vrethem M, Ernerudh J. Longitudinal interferon-beta effects in multiple sclerosis: differential regulation of IL-10 and IL-17A, while no sustained effects on IFN-gamma, IL-4 or IL-13. *Journal of the neurological sciences.* Feb 15 2013;325(1-2):79-85.

99. Lebrun C, Cohen M, Pignolet B, et al. Interleukin 17 alone is not a discriminant biomarker in early demyelinating spectrum disorders. *Journal of the neurological sciences.* Sep 15 2016;368:334-336.

100. Lomakin Y, Belogurov A, Jr., Glagoleva I, et al. Administration of Myelin Basic Protein Peptides Encapsulated in Mannosylated Liposomes Normalizes Level of Serum TNF-alpha and IL-2 and Chemoattractants CCL2 and CCL4 in Multiple Sclerosis Patients. *Mediators of inflammation.* 2016;2016:2847232.

101. Losy J, Michalowska-Wender G. In vivo effect of interferon-beta 1a on interleukin-12 and TGF-beta(1) cytokines in patients with relapsing-remitting multiple sclerosis. *Acta neurologica Scandinavica.* Jul 2002;106(1):44-46.

102. Losy J, Michalowska-Wender G, Wender M. Interleukin 12 and interleukin 10 are affected differentially by treatment of multiple sclerosis with glatiramer acetate (Copaxone). *Folia neuropathologica.* 2002;40(4):173-175.

103. Lund BT, Ashikian N, Ta HQ, et al. Increased CXCL8 (IL-8) expression in Multiple Sclerosis. *Journal of neuroimmunology.* Oct 2004;155(1-2):161-171.

104. Lundstrom W, Hermanrud C, Sjostrand M, et al. Interferon beta treatment of multiple sclerosis increases serum interleukin-7. *Multiple sclerosis.* Nov 2014;20(13):1727-1736.

105. Lus G, Di Biase G, Fratta M, Maniscalco G, Cotrufo R. Tumor necrosis factor-alpha and insulin-like growth factor-1 levels in patients with relapsing-remitting multiple sclerosis receiving interferon-beta1a. *Journal of interferon & cytokine research : the official journal of the International Society for Interferon and Cytokine Research.* May 2009;29(5):255-261.

106. Mahad DJ, Howell SJ, Woodroofe MN. Expression of chemokines in the CSF and correlation with clinical disease activity in patients with multiple sclerosis. *Journal of neurology, neurosurgery, and psychiatry.* Apr 2002;72(4):498-502.

107. Maimone D, Gregory S, Arnason BG, Reder AT. Cytokine levels in the cerebrospinal fluid and serum of patients with

multiple sclerosis. *Journal of neuroimmunology.* Apr 1991;32(1):67-74.

108. Malekzadeh A, Twaalfhoven H, Wijnstok NJ, Killestein J, Blankenstein MA, Teunissen CE. Comparison of multiplex platforms for cytokine assessments and their potential use for biomarker profiling in multiple sclerosis. *Cytokine.* Mar 2017;91:145-152.

109. Malmestrom C, Andersson BA, Haghighi S, Lycke J. IL-6 and CCL2 levels in CSF are associated with the clinical course of MS: implications for their possible immunopathogenic roles. *Journal of neuroimmunology.* Jun 2006;175(1-2):176-182.

110. Martinez-Caceres EM, Espejo C, Brieva L, et al. Expression of chemokine receptors in the different clinical forms of multiple sclerosis. *Multiple sclerosis.* Oct 2002;8(5):390-395.

111. Martins TB, Rose JW, Jaskowski TD, et al. Analysis of proinflammatory and anti-inflammatory cytokine serum concentrations in patients with multiple sclerosis by using a multiplexed immunoassay. *American journal of clinical pathology.* Nov 2011;136(5):696-704.

112. Matejcikova Z, Mares J, Prikrylova Vranova H, et al. Cerebrospinal fluid inflammatory markers in patients with multiple sclerosis: a pilot study. *Journal of neural transmission.* Feb 2015;122(2):273-277.

113. Matejcikova Z, Mares J, Sladkova V, et al. Cerebrospinal fluid and serum levels of interleukin-8 in patients with multiple sclerosis and its correlation with Q-albumin. *Multiple sclerosis and related disorders.* May 2017;14:12-15.

114. Matsushita T, Tateishi T, Isobe N, et al. Characteristic cerebrospinal fluid cytokine/chemokine profiles in neuromyelitis optica, relapsing remitting or primary progressive multiple sclerosis. *PloS one.* 2013;8(4):e61835.

115. Mellergard J, Edstrom M, Vrethem M, Ernerudh J, Dahle C. Natalizumab treatment in multiple sclerosis: marked decline of chemokines and cytokines in cerebrospinal fluid. *Multiple sclerosis.* Feb 2010;16(2):208-217.

116. Mellergard J, Tisell A, Dahlqvist Leinhard O, et al. Association between change in normal appearing white matter metabolites and intrathecal inflammation in natalizumab-treated multiple sclerosis. *PloS one.* 2012;7(9):e44739.

117. Michalopoulou M, Nikolaou C, Tavernarakis A, et al. Soluble interleukin-6 receptor (sIL-6R) in cerebrospinal fluid of patients with inflammatory and non inflammatory neurological diseases. *Immunology letters.* Jul 15 2004;94(3):183-189.

118. Michalowska-Wender G, Losy J, Biernacka-Lukanty J, Wender M. Impact of methylprednisolone treatment on the expression of macrophage inflammatory protein 3alpha and B lymphocyte chemoattractant in serum of multiple sclerosis patients. *Pharmacological reports : PR.* Jul-Aug 2008;60(4):549-554.

119. Mikulkova Z, Praksova P, Stourac P, Bednarik J, Michalek J. Imbalance in T-cell and cytokine profiles in patients with relapsing-remitting multiple sclerosis. *Journal of the neurological sciences.* Jan 15 2011;300(1-2):135-141.

120. Miljkovic D, Drulovic J, Trajkovic V, et al. Nitric oxide metabolites and interleukin-6 in cerebrospinal fluid from multiple sclerosis patients. *European journal of neurology.* Jul 2002;9(4):413-418.

121. Milosevic E, Dujmovic I, Markovic M, et al. Higher expression of IL-12Rbeta2 is associated with lower risk of relapse in relapsing-remitting multiple sclerosis patients on interferon-beta1b therapy during 3-year follow-up. *Journal of neuroimmunology.* Oct 15 2015;287:64-70.

122. Mirandola SR, Hallal DE, Farias AS, et al. Interferon-beta modifies the peripheral blood cell cytokine secretion in patients with multiple sclerosis. *International immunopharmacology.* Jul 2009;9(7-8):824-830.

123. Mori F, Nistico R, Nicoletti CG, et al. RANTES correlates with inflammatory activity and synaptic excitability in multiple sclerosis. *Multiple sclerosis.* Oct 2016;22(11):1405-1412.

124. Morsaljahan Z, Rafiei A, Valadan R, Abedini M, Pakseresht M, Khajavi R. Association between interleukin-32 polymorphism and multiple sclerosis. *Journal of the neurological sciences.* Aug 15 2017;379:144-150.

125. Mouzaki A, Rodi M, Dimisianos N, et al. Immune Parameters That Distinguish Multiple Sclerosis Patients from Patients with Other Neurological Disorders at Presentation. *PloS one.* 2015;10(8):e0135434.

126. Muls N, Nasr Z, Dang HA, Sindic C, van Pesch V. IL-22, GM-CSF and IL-17 in peripheral CD4+ T cell subpopulations

during multiple sclerosis relapses and remission. Impact of corticosteroid therapy. *PloS one.* 2017;12(3):e0173780.

127. Musabak U, Demirkaya S, Genc G, Ilikci RS, Odabasi Z. Serum adiponectin, TNF-alpha, IL-12p70, and IL-13 levels in multiple sclerosis and the effects of different therapy regimens. *Neuroimmunomodulation.* 2011;18(1):57-66.

128. Naderi S, Hejazi Z, Shajarian M, Alsahebfosoul F, Etemadifar M, Sedaghat N. IL-27 plasma level in relapsing remitting multiple sclerosis subjects: The double-faced cytokine. *Journal of immunoassay & immunochemistry.* 2016;37(6):659-670.

129. Napolitano M, Bruno A, Mastrangelo D, et al. Endovascular treatment of chronic cerebro spinal venous insufficiency in patients with multiple sclerosis modifies circulating markers of endothelial dysfunction and coagulation activation: a prospective study. *Blood coagulation & fibrinolysis : an international journal in haemostasis and thrombosis.* Oct 2014;25(7):716-720.

130. Narikawa K, Fujihara K, Misu T, et al. CSF-chemokines in HTLV-I-associated myelopathy: CXCL10 up-regulation and therapeutic effect of interferon-alpha. *Journal of neuroimmunology.* Feb 2005;159(1-2):177-182.

131. Nicoletti F, Di Marco R, Patti F, et al. Blood levels of transforming growth factor-beta 1 (TGF-beta1) are elevated in both relapsing remitting and chronic progressive multiple sclerosis (MS) patients and are further augmented by treatment with interferon-beta 1b (IFN-beta1b). *Clinical and experimental immunology.* Jul 1998;113(1):96-99.

132. Nicoletti F, Patti F, Cocuzza C, et al. Elevated serum levels of interleukin-12 in chronic progressive multiple sclerosis. *Journal of neuroimmunology.* Oct 1996;70(1):87-90.

133. Nicoletti F, Patti F, DiMarco R, et al. Circulating serum levels of IL-1ra in patients with relapsing remitting multiple sclerosis are normal during remission phases but significantly increased either during exacerbations or in response to IFN-beta treatment. *Cytokine.* May 1996;8(5):395-400.

134. Niedziela N, Adamczyk-Sowa M, Niedziela JT, et al. Assessment of Serum Nitrogen Species and Inflammatory Parameters in Relapsing-Remitting Multiple Sclerosis Patients Treated with Different Therapeutic Approaches. *BioMed research international.* 2016;2016:4570351.

135. Nischwitz S, Faber H, Samann PG, et al. Interferon beta-1a reduces increased interleukin-16 levels in multiple sclerosis patients. *Acta neurologica Scandinavica.* Jul 2014;130(1):46-52.

136. Noroozi S, Meimand HAE, Arababadi MK, Nakhaee N, Asadikaram G. The Effects of IFN-beta 1a on the Expression of Inflammasomes and Apoptosis-Associated Speck-Like Proteins in Multiple Sclerosis Patients. *Molecular neurobiology.* May 2017;54(4):3031-3037.

137. Novakova L, Axelsson M, Khademi M, et al. Cerebrospinal fluid biomarkers of inflammation and degeneration as measures of fingolimod efficacy in multiple sclerosis. *Multiple sclerosis.* Jan 2017;23(1):62-71.

138. Novakova L, Axelsson M, Khademi M, et al. Cerebrospinal fluid biomarkers as a measure of disease activity and treatment efficacy in relapsing-remitting multiple sclerosis. *Journal of neurochemistry.* Apr 2017;141(2):296-304.

139. Obradovic D, Kataranovski M, Dincic E, Obradovic S, Colic M. Tumor necrosis factor-alfa and interleukin-4 in cerbrospinal fluid and plasma in different clinical forms of multiple sclerosis. *Vojnosanitetski pregled.* Feb 2012;69(2):151-156.

140. Oliveira SR, Kallaur AP, Lopes J, et al. Insulin resistance, atherogenicity, and iron metabolism in multiple sclerosis with and without depression: Associations with inflammatory and oxidative stress biomarkers and uric acid. *Psychiatry research.* Apr 2017;250:113-120.

141. Oliveira SR, Kallaur AP, Reiche EMV, et al. Albumin and Protein Oxidation are Predictors that Differentiate Relapsing-Remitting from Progressive Clinical Forms of Multiple Sclerosis. *Molecular neurobiology.* May 2017;54(4):2961-2968.

142. Orefice NS, Alhouayek M, Carotenuto A, et al. Oral Palmitoylethanolamide Treatment Is Associated with Reduced Cutaneous Adverse Effects of Interferon-beta1a and Circulating Proinflammatory Cytokines in Relapsing-Remitting Multiple Sclerosis. *Neurotherapeutics : the journal of the American Society for Experimental NeuroTherapeutics.* Apr 2016;13(2):428-438.

143. Orhan G, Eruyar E, Mungan SO, Ak F, Karahalil B. The association of IL-18 gene promoter polymorphisms and the levels of serum IL-18 on the risk of multiple sclerosis. *Clinical neurology and neurosurgery.* Jul 2016;146:96-101.
144. Padberg F, Feneberg W, Schmidt S, et al. CSF and serum levels of soluble interleukin-6 receptors (sIL-6R and sgp130), but not of interleukin-6 are altered in multiple sclerosis. *Journal of neuroimmunology.* Oct 29 1999;99(2):218-223.
145. Paroni M, Maltese V, De Simone M, et al. Recognition of viral and self-antigens by TH1 and TH1/TH17 central memory cells in patients with multiple sclerosis reveals distinct roles in immune surveillance and relapses. *The Journal of allergy and clinical immunology.* Sep 2017;140(3):797-808.
146. Pashenkov M, Soderstrom M, Link H. Secondary lymphoid organ chemokines are elevated in the cerebrospinal fluid during central nervous system inflammation. *Journal of neuroimmunology.* Feb 2003;135(1-2):154-160.
147. Perriard G, Mathias A, Enz L, et al. Interleukin-22 is increased in multiple sclerosis patients and targets astrocytes. *Journal of neuroinflammation.* Jun 16 2015;12:119.
148. Piazza F, DiFrancesco JC, Fusco ML, et al. Cerebrospinal fluid levels of BAFF and APRIL in untreated multiple sclerosis. *Journal of neuroimmunology.* Mar 30 2010;220(1-2):104-107.
149. Piccio L, Naismith RT, Trinkaus K, et al. Changes in B- and T-lymphocyte and chemokine levels with rituximab treatment in multiple sclerosis. *Archives of neurology.* Jun 2010;67(6):707-714.
150. Polachini CR, Spanevello RM, Casali EA, et al. Alterations in the cholinesterase and adenosine deaminase activities and inflammation biomarker levels in patients with multiple sclerosis. *Neuroscience.* Apr 25 2014;266:266-274.
151. Puthenparampil M, Federle L, Miante S, et al. BAFF Index and CXCL13 levels in the cerebrospinal fluid associate respectively with intrathecal IgG synthesis and cortical atrophy in multiple sclerosis at clinical onset. *Journal of neuroinflammation.* Jan 17 2017;14(1):11.
152. Puthenparampil M, Miante S, Federle L, et al. BAFF is decreased in the cerebrospinal fluid of multiple sclerosis at clinical onset. *Journal of neuroimmunology.* Aug 15 2016;297:63-67.
153. Ragheb S, Li Y, Simon K, et al. Multiple sclerosis: BAFF and CXCL13 in cerebrospinal fluid. *Multiple sclerosis.* Jul 2011;17(7):819-829.
154. Ramirez-Ramirez V, Macias-Islas MA, Ortiz GG, et al. Efficacy of fish oil on serum of TNF alpha , IL-1 beta , and IL-6 oxidative stress markers in multiple sclerosis treated with interferon beta-1b. *Oxidative medicine and cellular longevity.* 2013;2013:709493.
155. Ramos-Cejudo J, Oreja-Guevara C, Stark Aroeira L, Rodriguez de Antonio L, Chamorro B, Diez-Tejedor E. Treatment with natalizumab in relapsing-remitting multiple sclerosis patients induces changes in inflammatory mechanism. *Journal of clinical immunology.* Aug 2011;31(4):623-631.
156. Reale M, Di Bari M, Di Nicola M, et al. Nicotinic receptor activation negatively modulates pro-inflammatory cytokine production in multiple sclerosis patients. *International immunopharmacology.* Nov 2015;29(1):152-157.
157. Rentzos M, Cambouri C, Rombos A, et al. IL-15 is elevated in serum and cerebrospinal fluid of patients with multiple sclerosis. *Journal of the neurological sciences.* Feb 15 2006;241(1-2):25-29.
158. Rentzos M, Nikolaou C, Rombos A, et al. Circulating interleukin-15 and RANTES chemokine in MS patients: effect of treatment with methylprednisolone in patients with relapse. *Neurological research.* Sep 2010;32(7):684-689.
159. Rentzos M, Nikolaou C, Rombos A, et al. Effect of treatment with methylprednisolone on the serum levels of IL-12, IL-10 and CCL2 chemokine in patients with multiple sclerosis in relapse. *Clinical neurology and neurosurgery.* Dec 2008;110(10):992-996.
160. Rentzos M, Nikolaou C, Rombos A, Voumvourakis K, Segditsa I, Papageorgiou C. Tumour necrosis factor alpha is elevated in serum and cerebrospinal fluid in multiple sclerosis and inflammatory neuropathies. *Journal of neurology.* Feb 1996;243(2):165-170.
161. Rollnik JD, Sindern E, Schweppe C, Malin JP. Biologically active TGF-beta 1 is increased in cerebrospinal fluid while it is reduced in serum in multiple sclerosis patients. *Acta neurologica Scandinavica.* Aug 1997;96(2):101-105.

162. Romme Christensen J, Bornsen L, Khademi M, et al. CSF inflammation and axonal damage are increased and correlate in progressive multiple sclerosis. *Multiple sclerosis.* Jun 2013;19(7):877-884.

163. Rossi S, Mancino R, Bergami A, et al. Potential role of IL-13 in neuroprotection and cortical excitability regulation in multiple sclerosis. *Multiple sclerosis.* Nov 2011;17(11):1301-1312.

164. Rossi S, Motta C, Studer V, et al. Tumor necrosis factor is elevated in progressive multiple sclerosis and causes excitotoxic neurodegeneration. *Multiple sclerosis.* Mar 2014;20(3):304-312.

165. Rossi S, Motta C, Studer V, et al. Subclinical central inflammation is risk for RIS and CIS conversion to MS. *Multiple sclerosis.* Oct 2015;21(11):1443-1452.

166. Rudick RA, Barna BP. Serum interleukin 2 and soluble interleukin 2 receptor in patients with multiple sclerosis who are experiencing severe fatigue. *Archives of neurology.* Mar 1990;47(3):254-255.

167. Ruocco G, Rossi S, Motta C, et al. T helper 9 cells induced by plasmacytoid dendritic cells regulate interleukin-17 in multiple sclerosis. *Clinical science.* Aug 2015;129(4):291-303.

168. Sadowska-Bartosz I, Adamczyk-Sowa M, Galiniak S, Mucha S, Pierzchala K, Bartosz G. Oxidative modification of serum proteins in multiple sclerosis. *Neurochemistry international.* Nov 2013;63(5):507-516.

169. Salama HH, Kolar OJ, Zang YC, Zhang J. Effects of combination therapy of beta-interferon 1a and prednisone on serum immunologic markers in patients with multiple sclerosis. *Multiple sclerosis.* Feb 2003;9(1):28-31.

170. Salehi M, Bagherpour B, Shayghannejad V, Mohebi F, Jafari R. Th1, Th2 and Th17 Cytokine Profile in Patients with Multiple Sclerosis Following Treatment with Rapamycin. *Iranian journal of immunology : IJI.* Jun 2016;13(2):141-147.

171. Salmaggi A, Dufour A, Eoli M, et al. Low serum interleukin-10 levels in multiple sclerosis: further evidence for decreased systemic immunosuppression? *Journal of neurology.* Jan 1996;243(1):13-17.

172. Sanoobar M, Eghtesadi S, Azimi A, et al. Coenzyme Q10 supplementation ameliorates inflammatory markers in patients with multiple sclerosis: a double blind, placebo, controlled randomized clinical trial. *Nutritional neuroscience.* May 2015;18(4):169-176.

173. Saruhan-Direskeneli G, Yentur SP, Akman-Demir G, Isik N, Serdaroglu P. Cytokines and chemokines in neuro-Behcet's disease compared to multiple sclerosis and other neurological diseases. *Journal of neuroimmunology.* Dec 2003;145(1-2):127-134.

174. Sayad A. The association of -330 interleukin-2 gene polymorphism and HLA-DR15 allele in Iranian patients with multiple sclerosis. *International journal of immunogenetics.* Aug 2014;41(4):330-334.

175. Sayad A, Movafagh A. The association of -330 interleukin-2 gene polymorphism with its plasma concentration in Iranian multiple sclerosis patients. *Scientifica.* 2014;2014:724653.

176. Scalabrino G, Galimberti D, Mutti E, et al. Loss of epidermal growth factor regulation by cobalamin in multiple sclerosis. *Brain research.* May 28 2010;1333:64-71.

177. Seledtsova GV, Ivanova IP, Shishkov AA, Seledtsov VI. Immune responses to polyclonal T-cell vaccination in patients with progressive multiple sclerosis. *Journal of immunotoxicology.* Nov 2016;13(6):879-884.

178. Sellebjerg F, Bornsen L, Khademi M, et al. Increased cerebrospinal fluid concentrations of the chemokine CXCL13 in active MS. *Neurology.* Dec 8 2009;73(23):2003-2010.

179. Sellner J, Greeve I, Findling O, et al. Effect of interferon-beta and atorvastatin on Th1/Th2 cytokines in multiple sclerosis. *Neurochemistry international.* Jul 2008;53(1-2):17-21.

180. Sexton M, Cudaback E, Abdullah RA, et al. Cannabis use by individuals with multiple sclerosis: effects on specific immune parameters. *Inflammopharmacology.* Oct 2014;22(5):295-303.

181. Shajarian M, Alsahebfosoul F, Etemadifar M, et al. IL-23 plasma level measurement in relapsing remitting multiple sclerosis (RRMS) patients compared to healthy subjects. *Immunological investigations.* 2015;44(1):36-44.

182. Sharief MK, Hentges R, Thompson EJ. The relationship of interleukin-2 and soluble interleukin-2 receptors to

intrathecal immunoglobulin synthesis in patients with multiple sclerosis. *Journal of neuroimmunology.* Apr 1991;32(1):43-51.

183. Shu Y, Li R, Qiu W, et al. Association of serum gamma-glutamyltransferase and C-reactive proteins with neuromyelitis optica and multiple sclerosis. *Multiple sclerosis and related disorders.* Nov 2017;18:65-70.

184. Sorensen TL, Ransohoff RM, Strieter RM, Sellebjerg F. Chemokine CCL2 and chemokine receptor CCR2 in early active multiple sclerosis. *European journal of neurology.* Jul 2004;11(7):445-449.

185. Sorensen TL, Tani M, Jensen J, et al. Expression of specific chemokines and chemokine receptors in the central nervous system of multiple sclerosis patients. *The Journal of clinical investigation.* Mar 1999;103(6):807-815.

186. Stepien A, Chalimoniuk M, Lubina-Dabrowska N, Chrapusta SJ, Galbo H, Langfort J. Effects of interferon beta-1a and interferon beta-1b monotherapies on selected serum cytokines and nitrite levels in patients with relapsing-remitting multiple sclerosis: a 3-year longitudinal study. *Neuroimmunomodulation.* 2013;20(4):213-222.

187. Su N, Shi SX, Zhu X, Borazanci A, Shi FD, Gan Y. Interleukin-7 expression and its effect on natural killer cells in patients with multiple sclerosis. *Journal of neuroimmunology.* Nov 15 2014;276(1-2):180-186.

188. Sumita Y, Murakawa Y, Sugiura T, Wada Y, Nagai A, Yamaguchi S. Elevated BAFF levels in the cerebrospinal fluid of patients with neuro-Behcet's disease: BAFF is correlated with progressive dementia and psychosis. *Scandinavian journal of immunology.* Jun 2012;75(6):633-640.

189. Szczucinski A, Losy J. CCL5, CXCL10 and CXCL11 chemokines in patients with active and stable relapsing-remitting multiple sclerosis. *Neuroimmunomodulation.* 2011;18(1):67-72.

190. Tanaka M, Matsushita T, Tateishi T, et al. Distinct CSF cytokine/chemokine profiles in atopic myelitis and other causes of myelitis. *Neurology.* Sep 23 2008;71(13):974-981.

191. Tang SC, Fan XH, Pan QM, Sun QS, Liu Y. Decreased expression of IL-27 and its correlation with Th1 and Th17 cells in progressive multiple sclerosis. *Journal of the neurological sciences.* Jan 15 2015;348(1-2):174-180.

192. Tao Y, Zhang X, Chopra M, et al. The role of endogenous IFN-beta in the regulation of Th17 responses in patients with relapsing-remitting multiple sclerosis. *Journal of immunology.* Jun 15 2014;192(12):5610-5617.

193. Tawfik TZ, Gad AH, Mehaney DA, et al. Interleukins 17 and 10 in a sample of Egyptian relapsing remitting multiple sclerosis patients. *Journal of the neurological sciences.* Oct 15 2016;369:36-38.

194. Tejera-Alhambra M, Casrouge A, de Andres C, et al. Plasma biomarkers discriminate clinical forms of multiple sclerosis. *PloS one.* 2015;10(6):e0128952.

195. Tiumentseva M, Morozova V, Zakabunin A, et al. Use of the VH6-1 gene segment to code for anti-interleukin-18 autoantibodies in multiple sclerosis. *Immunogenetics.* Apr 2016;68(4):237-246.

196. Toghianifar N, Ashtari F, Zarkesh-Esfahani SH, Mansourian M. Effect of high dose vitamin D intake on interleukin-17 levels in multiple sclerosis: a randomized, double-blind, placebo-controlled clinical trial. *Journal of neuroimmunology.* Aug 15 2015;285:125-128.

197. Tomioka R, Hamaguchi K, Ohno R, et al. Elevated interleukin 2 levels in serum and cerebrospinal fluid of patients with relapsing-remitting multiple sclerosis. *Annals of the New York Academy of Sciences.* Apr 15 1992;650:347-350.

198. Tong Y, Yang T, Wang J, et al. Elevated Plasma Chemokines for Eosinophils in Neuromyelitis Optica Spectrum Disorders during Remission. *Frontiers in neurology.* 2018;9:44.

199. Trenova AG, Slavov GS, Manova MG, et al. Alterations in serum levels of IL-17 in contrast to TNF-alpha correspond to disease-modifying treatment in relapsing-remitting multiple sclerosis. *Scandinavian journal of clinical and laboratory investigation.* Jul 2017;77(4):283-288.

200. Trenova AG, Slavov GS, Manova MG, Kostadinova, II. Cytokines and disability in interferon-beta-1b treated and untreated women with multiple sclerosis. *Archives of medical research.* Aug 2014;45(6):495-500.

201. Trotter JL, Clifford DB, McInnis JE, et al. Correlation of immunological studies and disease progression in chronic progressive multiple sclerosis. *Annals of neurology.* Feb 1989;25(2):172-178.

202. Trotter JL, Collins KG, van der Veen RC. Serum cytokine levels in chronic progressive multiple sclerosis: interleukin-2 levels parallel tumor necrosis factor-alpha levels. *Journal of neuroimmunology.* Jul 1991;33(1):29-36.

203. Trotter JL, van der Veen RC, Clifford DB. Serial studies of serum interleukin-2 in chronic progressive multiple sclerosis patients: occurrence of 'bursts' and effect of cyclosporine. *Journal of neuroimmunology.* Jun 1990;28(1):9-14.

204. Tsukada N, Matsuda M, Miyagi K, Yanagisawa N. Soluble CD4 and CD8 in the peripheral blood of patients with multiple sclerosis and HTLV-1-associated myelopathy. *Journal of neuroimmunology.* Dec 1991;35(1-3):285-293.

205. Tsukada N, Miyagi K, Matsuda M, Yanagisawa N, Yone K. Tumor necrosis factor and interleukin-1 in the CSF and sera of patients with multiple sclerosis. *Journal of the neurological sciences.* Aug 1991;104(2):230-234.

206. Tumani H, Kassubek J, Hijazi M, et al. Patterns of TH1/TH2 cytokines predict clinical response in multiple sclerosis patients treated with glatiramer acetate. *European neurology.* 2011;65(3):164-169.

207. Uzawa A, Mori M, Arai K, et al. Cytokine and chemokine profiles in neuromyelitis optica: significance of interleukin-6. *Multiple sclerosis.* Dec 2010;16(12):1443-1452.

208. Valenzuela RM, Kaufman M, Balashov KE, Ito K, Buyske S, Dhib-Jalbut S. Predictive cytokine biomarkers of clinical response to glatiramer acetate therapy in multiple sclerosis. *Journal of neuroimmunology.* Nov 15 2016;300:59-65.

209. Vrethem M, Kvarnstrom M, Stenstam J, et al. Cytokine mapping in cerebrospinal fluid and blood in multiple sclerosis patients without oligoclonal bands. *Multiple sclerosis.* May 2012;18(5):669-673.

210. Wang H, Wang K, Zhong X, et al. Notable increased cerebrospinal fluid levels of soluble interleukin-6 receptors in neuromyelitis optica. *Neuroimmunomodulation.* 2012;19(5):304-308.

211. Wang KC, Lee CL, Chen SY, et al. Distinct serum cytokine profiles in neuromyelitis optica and multiple sclerosis. *Journal of interferon & cytokine research : the official journal of the International Society for Interferon and Cytokine Research.* Feb 2013;33(2):58-64.

212. Wang S, Yang T, Wan J, Zhang Y, Fan Y. Elevated C-X-C motif ligand 13 and B-cell-activating factor levels in neuromyelitis optica during remission. *Brain and behavior.* Apr 2017;7(4):e00648.

213. Wang Y, Zhou Y, Sun X, et al. Cytokine and Chemokine Profiles in Patients with Neuromyelitis Optica Spectrum Disorder. *Neuroimmunomodulation.* 2016;23(5-6):352-358.

214. Weller M, Stevens A, Sommer N, Melms A, Dichgans J, Wietholter H. Comparative analysis of cytokine patterns in immunological, infectious, and oncological neurological disorders. *Journal of the neurological sciences.* Aug 1991;104(2):215-221.

215. Wen SR, Liu GJ, Feng RN, et al. Increased levels of IL-23 and osteopontin in serum and cerebrospinal fluid of multiple sclerosis patients. *Journal of neuroimmunology.* Mar 2012;244(1-2):94-96.

216. Wiesemann E, Klatt J, Wenzel C, Heidenreich F, Windhagen A. Correlation of serum IL-13 and IL-5 levels with clinical response to Glatiramer acetate in patients with multiple sclerosis. *Clinical and experimental immunology.* Sep 2003;133(3):454-460.

217. Wing AC, Hygino J, Ferreira TB, et al. Interleukin-17- and interleukin-22-secreting myelin-specific CD4(+) T cells resistant to corticoids are related with active brain lesions in multiple sclerosis patients. *Immunology.* Feb 2016;147(2):212-220.

218. Witkowska AM, Socha K, Kochanowicz J, et al. Serum Levels of Biomarkers of Immune Activation and Associations With Neurological Impairment in Relapsing-Remitting Multiple Sclerosis Patients During Remission. *Biological research for nursing.* Jan 2016;18(1):113-119.

219. Wu A, Zhong X, Wang H, et al. Cerebrospinal fluid IL-21 levels in Neuromyelitis Optica and multiple sclerosis. *The Canadian journal of neurological sciences. Le journal canadien des sciences neurologiques.* Nov 2012;39(6):813-820.

220. Wullschleger A, Kapina V, Molnarfi N, et al. Cerebrospinal fluid interleukin-6 in central nervous system inflammatory diseases. *PloS one.* 2013;8(8):e72399.

221. Xu W, Li R, Dai Y, et al. IL-22 secreting CD4+ T cells in the patients with neuromyelitis optica and multiple sclerosis.

Journal of neuroimmunology. Aug 15 2013;261(1-2):87-91.

222. Yang T, Wang S, Zheng Q, et al. Increased plasma levels of epithelial neutrophil-activating peptide 78/CXCL5 during the remission of Neuromyelitis optica. *BMC neurology.* Jul 11 2016;16:96.

223. Yeung D, Ciotti S, Purushothama S, et al. Evaluation of highly sensitive immunoassay technologies for quantitative measurements of sub-pg/mL levels of cytokines in human serum. *Journal of immunological methods.* Oct 2016;437:53-63.

224. Zhang X, Tao Y, Chopra M, et al. IL-11 Induces Th17 Cell Responses in Patients with Early Relapsing-Remitting Multiple Sclerosis. *Journal of immunology.* Jun 1 2015;194(11):5139-5149.

225. Zhen J, Yuan J, Fu Y, et al. IL-22 promotes Fas expression in oligodendrocytes and inhibits FOXP3 expression in T cells by activating the NF-kappaB pathway in multiple sclerosis. *Molecular immunology.* Feb 2017;82:84-93.

226. Zhong X, Wang H, Dai Y, et al. Cerebrospinal fluid levels of CXCL13 are elevated in neuromyelitis optica. *Journal of neuroimmunology.* Dec 15 2011;240-241:104-108.
